# Discovery and characterization of heterogeneous and multipotent fibroblast populations isolated from excised cleft lip tissue

**DOI:** 10.1186/s13287-022-03154-x

**Published:** 2022-09-08

**Authors:** Ludovica Parisi, Silvia Rihs, Giorgio C. La Scala, Isabelle Schnyder, Christos Katsaros, Martin Degen

**Affiliations:** 1 Laboratory for Oral Molecular Biology, Department of Orthodontics and Dentofacial Orthopedics, University of Bern, Bern, Switzerland; 2 Division of Pediatric Surgery, Department of Pediatrics, University Hospital of Geneva, Geneva, Switzerland; 3 University Clinic for Pediatric Surgery, Bern University Hospital, Bern, Switzerland

**Keywords:** Cleft lip/palate, Fibroblasts, Mesenchymal stem cells, Single cell clonal analysis

## Abstract

**Background:**

Regularly discarded lip tissue obtained from corrective surgeries to close the cleft lip represents an easily accessible and rich source for the isolation of primary fibroblasts. Primary fibroblasts have been described to show compelling similarities to mesenchymal stem cells (MSCs). Hence, cleft lip and palate (CLP) lip-derived fibroblasts could be thought as an intriguing cell source for personalized regenerative therapies in CLP-affected patients.

**Methods:**

Initially, we thoroughly characterized the fibroblastic nature of the lip-derived mesenchymal outgrowths by molecular and functional assays. Next, we compared their phenotype and genotype to that of bone marrow-mesenchymal stem cells (BM-MSCs) and of human lung-derived fibroblasts WI38, by assessing their morphology, surface marker expression, trilineage differentiation potential, colony-forming (CFU) capacity, and immunomodulation property. Finally, to better decipher the heterogeneity of our CLP cultures, we performed a single cell clonal analysis and tested expanded clones for surface marker expression, as well as osteogenic and CFU potential.

**Results:**

We identified intriguingly similar phenotypic and genotypic properties between CLP lip fibroblasts and BM-MSCs, which makes them distinct from WI38. Furthermore, our own data in combination with the complex anatomy of the lip tissue indicated heterogeneity in our CLP cultures. Using a clonal analysis, we discovered single cell-derived clones with increased levels of the MSC markers CD106 and CD146 and clones with variabilities in their commitment to differentiate into bone-forming cells and in their potential to form single cell-derived colonies. However, we were not able to gain clones possessing superior MSC-like capacities when compared to the heterogeneous parental CLP population. Additionally, all clones could still generate contractile forces and retained robust levels of the fibroblast specific marker FSP1, which was not detectable in BM-MSCs.

**Conclusions:**

Our results suggest that we isolate heterogeneous populations of fibroblasts from discarded CLP lip tissue, which show a prominently multipotent character in their entirety avoiding the need for elaborate subpopulation selections in vitro. These findings suggest that CLP lip fibroblasts might be a novel potential cell source for personalized regenerative medicine of clinical benefit for CLP patients.

**Supplementary Information:**

The online version contains supplementary material available at 10.1186/s13287-022-03154-x.

## Background

Cleft lip and palate (CLP) represents the most common congenital craniofacial malformation, affecting 1:750 newborns, and is caused by defective embryonic development of soft as well as hard facial tissues [1]. Current treatment strategies require surgical repair of the orofacial cleft within the first 2 years of life. Unfortunately, CLP repair turns out to be a more complex and comprehensive process, often demanding additional surgeries to correct potential associated problems during infancy and adolescence. These include improper dentition, speech and hearing difficulties, excessive scarring, and not optimal aesthetics [2, 3]. Yet, some of these secondary functional and/or aesthetical interventions can be challenging as often not enough tissue is locally available to repair the defect [4]. For instance, if the orofacial cleft extends into the front part of the alveolar bone, a bone augmentation procedure is required, which demands for yet an additional surgery in another anatomical region to harvest autogenous bone [5]. Therefore, less invasive strategies for fixing alveolar bone clefts are desired. Regenerative therapies might be such approaches as they have the potential to significantly lower the burden of CLP-affected patients by providing a compelling alternative to bone grafts.

Autologous mesenchymal stem cells (MSCs) offer promising opportunities to promote tissue regeneration. Accordingly, more than 1400 clinical trials are registered (www.clinicaltrials.gov, lastly accessed on July 31st, 2022), aiming to apply MSCs for various medical indications, including the handling of congenital craniofacial defects, such as interruption of the alveolar bone in CLP patients [6]. MSCs were initially identified in bone marrow [7] and since then have been described in many other adult and fetal tissues [8–13], making a precise and standardized characterization unavoidable. Accordingly, the International Society for Cellular Therapy proposed a set of three minimal criteria to define MSCs [14]. MSCs must (1) be plastic-adherent cells of spindle-like morphology, (2) express the cell surface markers CD73, CD90, and CD105, while being devoid of CD34, CD45, CD31, CD14, CD19, CD11b, CD79a, and HLA-DR, and (3) display a differentiation potential into bone-, fat-, and cartilage-forming cells. Additional MSC-related traits, such as an enhanced proliferative efficiency and immunomodulatory capacity, have been more recently identified [15–17]. Yet, the use of MSCs for tissue regeneration comes with some challenges, which slowed down their adoption in clinical settings. Firstly, isolation of MSCs requires an invasive procedure (i.e., bone marrow harvesting) involving general anesthesia, hospitalization, pain, and discomfort for the patient [18]. Secondly, the number of MSCs is estimated to be around 1/10^6^ nucleated cells in the adult bone marrow, which makes them a rare population [19, 20]. Consequently, in vitro expansion of MSCs is necessary for clinical use, which might increase the risk of MSCs to undergo an adaptive transformation or to acquire genomic instability as it has been observed for other stem cells [21–23]. Thirdly, MSCs are very sensitive to mechanical stress at the transplantation site and to biophysical parameters (e.g., oxygen levels, mechanical properties of the microenvironment) [24, 25]. Last but not least, it has been noted that the number of MSCs rapidly decreases with age (tenfold reduction from birth to teens) making MSC harvesting at young donor age crucially important [26]. Besides all these practical hurdles, long-term data on the safety of MSCs in clinical applications are also missing [27]. All these reasons justify the search for an alternative, more practical, and efficient cell source for MSCs.

Fibroblasts are mesenchymal cells, which play fundamental roles in the maintenance of tissue homeostasis by producing extracellular matrix (ECM) [28]. Historically, they have been considered as terminally differentiated cells, except for their potential to acquire a transient myofibroblast phenotype in response to tissue damage [29]. However, several recent studies are challenging this conservative dogma as compelling similarities between primary fibroblasts and MSCs have emerged. Indeed, in addition to be the master regulators of tissue homeostasis, fibroblasts have also been depicted as plastic-adherent, CD73-, CD90-, CD105-expressing cells, owning a trilineage differentiation capacity [21, 30–34]. Hence, the presence of these MSC-like properties, in combination with their easy accessibility due to their high abundance in tissue stroma, might promote primary fibroblasts as an alternative autologous cell source for MSCs in regenerative therapies. Unfortunately, a proper and robust comparison between MSCs and fibroblasts regarding all their ascribed activities is still missing and therefore highly warranted.

Recently, we have established a unique primary cranio-/orofacial-derived human cell bank, which includes stromal cells isolated from the lip tissue of CLP infants [35, 36]. We isolate these CLP patient cells from superfluous lip tissue reaching into the cleft, which has to be excised and is usually discarded during the corrective surgery to close the cleft lip at the age of 3–6 months. Our hypothesis is that CLP patient-derived fibroblasts may represent a viable and easily accessible source of autologous cells offering multipotent character.

A comparative analysis of various MSC-like traits between bone marrow-derived MSCs (BM-MSCs) and CLP lip-derived fibroblasts did not reveal any strong distinguishing features for the two cell populations. However, we noted a prominent heterogeneity present in our CLP lip fibroblasts, which might be derived from the complex lip anatomy and/or from the existence of different fibroblast clusters within the dermis [37, 38]. To exclude the possibility of having subpopulations within our fibroblasts that possess better multipotent capacities compared to the heterogeneous parental strain, we performed a clonal analysis. Although the single cell-derived clones displayed variable MSC-like activities and marker expressions, we were not able to identify subpopulations possessing superior traits compared to the whole population. Importantly, we could not enrich for MSCs in our populations as all single cell-derived clones retained their fibroblast identity, as assessed by the capacity to generate contractile forces as well as by the expression of fibroblast markers, such as fibroblast-specific protein 1 (FSP1).

Collectively, we present data that promote discarded lip tissue from CLP patients as a rich source for the isolation of multipotent fibroblasts that could be used as an alternative to MSCs in regenerative therapies. Hence, our CLP living cell repository [36] might provide a relevant clinical benefit for CLP patients later in their lives if additional tissue is needed for secondary surgeries.

## Methods

### Ethics statement

This work was performed according to the Ethical Principles for Medical Research Involving Human Subjects as stated in the Declaration of Helsinki by the World Medical Association. Isolation of human cleft lip-derived as well as human foreskin cells has been approved by the Kantonale Ethikkommision of Bern, Switzerland (protocol number: 2017-01394). Written informed consent was obtained from the legal representative of the children.

### Histology and immunohistochemistry (IHC)

To prepare formalin-fixed and paraffin-embedded blocks, tissue samples were fixed in 10% formalin for 24 h at room temperature (RT). Afterward, samples were dehydrated with increasing alcohol concentrations, followed by changes in xylene, before paraffin embedding. Five- to six-µm sections were cut on a Reichert-Jung microtome (Leica Microsystems, Wetzlar, Germany) and stained with hematoxylin–eosin (H&E) or further processed for IHC.

IHC staining reactions were performed by an automated Leica BOND RX system (Leica Biosystems). Tris-buffer, pH 9.0 at 95 °C for 30 min was used for antigen retrieval. Specific binding of primary antibodies was visualized through a polymer-based visualizing system with horseradish peroxidase as the enzyme and the 3,3-diaminobenzidine as brown chromogen (BOND Polymer Refine Detection, Leica Biosystems) for 10 min at RT. Cell nuclei were counterstained with hematoxylin and slides where mounted with Aquatex (Sigma-Aldrich, St. Louis, MO, USA).

Primary antibodies used for IHC: mouse monoclonal antibodies anti-E-cadherin (clone HECD-1, Thermo Fisher Scientific, Waltham, MA, USA), anti-Vimentin (clone 3B4), anti-CD31 (clone JC/70A), anti-HLA-DR (clone CR3/43, all from Dako Agilent, Santa Clara, CA, USA), and anti-skeletal myosin (clone MY-32, Sigma-Aldrich).

### Cell cultures

Using the explant culture technique, primary keratinocytes and fibroblasts were isolated and purified from CLP lip biopsies obtained from 3- to 6-month-old infants undergoing primary surgery to close the cleft lip at the Children’s Hospital, University of Bern [35, 36]. Fibroblasts were grown in Dulbecco’s modified Eagle’s medium (DMEM, Thermo Fisher Scientific) supplemented with 10% fetal calf serum (FCS, Sigma-Aldrich) and 1X penicillin and streptomycin (PenStrep, Thermo Fisher Scientific), while keratinocytes were grown in keratinocyte serum-free medium (KSFM, Thermo Fisher Scientific) containing 25 µg/ml bovine pituitary extract, 0.2 ng/ml epidermal growth factor, 0.4 mM CaCl_2_ and 1X PenStrep as described elsewhere [39]. Similarly, foreskin-derived fibroblasts were isolated from a biopsy obtained from a 7-year-old boy during routine circumcision at the Children’s Hospital, University of Bern. Details about non-commercial primary cells used in this study are reported in Table 1. Primary human BM-MSCs were purchased from ScienCell and cultured in MSC medium (ScienCell, Carlsbad, CA, USA). All experiments were performed with cultures from the third to the fifth passage. For low-serum cultures, after washing in phosphate-buffered saline (PBS) cells were starved in DMEM/0.3%FCS for 24 h before analysis. The human embryonic lung fibroblast WI38 cell line (a gift from Prof. Beat Trueb, DBMR, University of Bern, Switzerland) was cultured in DMEM/10%FCS/1XPenStrep, supplemented with 1X non-essential amino acids (Thermo Fisher Scientific). Cell pellets of C2C12 myoblasts, human umbilical vein endothelial cells (HUVEC, gift from Prof. Robert Rieben, DBMR, University of Bern, Switzerland), U937 (histiocytic lymphoma-derived human monocytes, gift from Prof. Stephan von Gunten, Institute of Pharmacology, University of Bern, Switzerland) and adipocytes (CLP-Ad, obtained during cell isolation from a premaxilla specimen of a CLP patient) were used as positive controls. Live images of the cells were taken with a Leica DMIL LED-inverted microscope (Leica Biosystems).

**Table 1:** List of the primary cells used

Sample	Cells	Donor sex	Donor age	Diagnosis
CLP-Ep	Keratinocytes	M	3 m	i CLP unilateral
CLP1	Fibroblasts	M	3 m	i CLP unilateral
CLP2	Fibroblasts	M	3 m	c CLP unilateral
CLP3	Fibroblasts	F	4 m	c CLP unilateral
CLP4	Fibroblasts	M	6 m	c CLP bilateral
CLP5	Fibroblasts	M	5 m	c CLP unilateral
Frsk-Fb	Fibroblasts	M	7 y	n.a.

M, male; F, female; m, months; y, years; i, incomplete; c, complete; n.a., not applicable

### Cell morphological analyses

For quantitative morphological analyses, cells were fixed in 4% paraformaldehyde (PFA, Grogg Chemie, Stettlen, Switzerland) for 20 min at RT, rinsed twice in PBS, and stained with 0.5% crystal violet (CV, Sigma-Aldrich) in 20% methanol for 20 min at RT on an orbital shaker. Excessive CV was removed by washing the dishes extensively with double-distilled water (ddH_2_O) before air-drying. Images were captured with an Olympus BX-51 phase microscope (Olympus Life Science Solution, Tokyo, Japan) and analyzed with the ImageJ software (https://imagej.nih.gov/ij/). Cell circularity (cc) was calculated as cc = 4*π*(*A*/*P*^2^) (*A*: cell area, *P*: cell perimeter).

### Immunofluorescence (IF)

For staining, cells were grown in 35-mm dishes containing four separate wells (Greiner Bio-One, Frickenhausen, Germany). Cells were fixed in 4% PFA, rinsed 3X in PBS, permeabilized with 0.1% Triton-X-100 (Sigma-Aldrich) for 5 min, blocked in 3% bovine serum albumin (Sigma-Aldrich) for 30 min and incubated with primary antibodies for 2 h at RT. Afterwards, cultures were washed 3X with PBS, incubated with fluorescent-labeled secondary antibodies (Molecular Probes, Thermo Fisher Scientific) and if needed with tetramethylrhodamine-phalloidin (Sigma-Aldrich) for 1 h at RT in the dark. Incubation was followed by 3X washes in PBS and one final rinse in ddH_2_O before being coverslip-mounted with the Vectashield Mounting Medium containing DAPI (Vector Laboratories, Burlingame, CA, USA).

To analyze ECM deposition, confluent cultures were decellularized with 20 mM ammonium hydroxide (Sigma-Aldrich) containing 0.5% Triton-X-100 for 30 min at 37 °C. After extensive washing with PBS, ECMs were fixed with 4% PFA and stained as described before.

Samples were analyzed under an Olympus BX-51 phase microscope equipped with fluorescence filters U-MWIBA3 for Alexa Fluor 488, U-MWIGA3 for Alexa Fluor 568, and U-MNUA2 for DAPI (Olympus Life Science Solution) detection.

Primary antibodies used: rabbit polyclonal antibodies anti-E-cadherin (20874-1-AP), anti-fibroblast-specific protein 1 (16105-1-AP, both from Proteintech, Manchester, UK) and anti-fibronectin [40], as well as mouse monoclonal antibodies anti-Vimentin (clone VI-10, A86652, Antibodies.com, Cambridge, UK) and anti-Vinculin (clone hVIN-1, V9131, Sigma-Aldrich).

### RNA extraction, cDNA synthesis, and quantitative real-time polymerase chain reaction (qPCR)

Total RNA from cells, micro-masses, or cell pellets was extracted using the innuPREP RNA Mini kit (Analytic Jena AG, Jena, Germany) according to their standard protocol. RNA concentration was measured with a NanoDrop 2000c (Thermo Fisher Scientific) and stored at − 80 °C until use.

cDNA was synthetized from 500 ng of total RNA using an Oligo(dT)_15_ primer and the M-MLV Reverse Transcriptase (both from Promega, Dübendorf, Switzerland).

Gene expression was analyzed by qPCR using the GoTaq® qPCR Master Mix (Promega) on a QuantStudio 3 instrument (Applied Biosystems, Thermo Fisher Scientific). Data analysis was performed applying the dC_*T*_ method when absolute mRNA normalized to *GAPDH* levels are reported, or by ddC_*T*_ method when absolute mRNA normalized to *GAPDH* is further referenced to a control sample set to 1.

The sequences of the qPCR primers used are listed in Additional file 1: Table S1 and were either taken from the PrimerBank database (http://pga.mgh.harvard.edu/primerbank/) or from the NCBI primer designing tool (http://ncbi.nlm.mih.gov/tools/primer-blast). All primer pairs were tested for specificity and efficiency using cDNA standard curves.

### Immunoblotting

Cell extracts were prepared in 1X RIPA buffer (10 mM Tris–HCl pH 8.0, 1 mM EDTA, 0.1% Na deoxycholate, 0.1% SDS, 1% NP40, 140 mM NaCl) supplemented with cOmplete Mini™ Protease Inhibitor cocktail and PhoSTOP EASYpack (both from Sigma-Aldrich). Protein concentrations were measured using a Bicinchoninic acid assay (Pierce, Thermo Fisher Scientific) following the manufacturer’s protocol. Conditioned media (CM) were prepared in DMEM/1XPenStrep supplemented with fibronectin-free 10% FCS [41] for 24 h, collected, centrifuged at 1000xg for 5 min and supernatants stored at − 80 °C until use.

Ten micrograms of total protein was diluted in sample loading buffer (62.6 mM Tris–HCl pH 6.8, 2% SDS, 10% glycerol, 0.01% bromophenol blue) containing 100 mM dithiothreitol (Sigma-Aldrich), boiled for 5 min at 95 °C, and separated under reducing conditions by SDS-PAGE. Proteins were blotted onto nitrocellulose or PVDF membranes (Sigma-Aldrich), which were subsequently stained with a 0.1% amido black solution (Merck, Burlington, MA, USA) to control for equal protein loading and blotting efficiency. Membranes were washed in Tris-buffered saline (TBS) pH 7.4, containing 0.05% Tween-20 (TBS-T), blocked in 5% skim milk in TBS-T, incubated with primary antibody overnight at 4 °C on a shaker, washed 3X in TBS-T, and incubated with horseradish peroxidase-conjugated anti-mouse or rabbit IgGs. After 3 more washes in TBS-T, blots were developed using the SuperSignal West Pico or Dura solutions (Pierce, Thermo Fisher Scientific) and scanned with an Imager Chemi Premium Instrument (VWR, Darmstadt, Germany). Primary antibodies used are the same as described above for IF in addition to the rabbit polyclonal anti-ß-Actin antibody (20536-1-AP, Proteintech).

### Collagen contraction assay

All solutions were cooled on ice before preparing collagen gels. Briefly, a 1.5 mg/ml collagen gel solution was prepared by sequentially adding 5.25 ml of Collagen R solution (2 mg/ml sterile rat tail collagen type I, SERVA Electrophoresis GmbH, Heidelberg, Germany), 0.7 ml 10XDMEM/1XPenStrep (Thermo Fisher Scientific), 0.7 ml sterile-filtered 0.44 M NaHCO_3_ (Sigma-Aldrich), 50 µl sterile-filtered 1.5 N NaOH (Sigma-Aldrich) and 0.35 ml 60% FCS in PBS. Four hundred µl of collagen gel solution was added per well of a 24-well plate and incubated for 30 min at 37 °C to allow gelation. Afterwards, collagen gels were seeded with 10^5^cells/well in DMEM/10%FCS/1XPenStrep. Cells were allowed to attach for 24 h, before the gels were carefully detached from the walls of the wells. The size of the gel was monitored for 8 h using an Olympus SZX7 stereomicroscope (Olympus Life Science Solution). Images were analyzed with ImageJ.

### Fluorescence-activated cell sorting (FACS) analysis

Expression of cell surface and intracellular markers was assessed using a flow cytometer (BD FACS SORP LSR II, BD Biosciences, Allschwil, Switzerland) and analyzed with the FlowJo software (BD Biosciences). For cell surface marker staining, cells were detached from tissue culture dishes using accutase (Thermo Fisher Scientific), resuspended in PBS/5%FCS at a final concentration of 10^7^cells/ml, and blocked with Fc block (BioLegend, San Diego, CA, USA) for 10 min at RT. Afterwards, cell suspensions were incubated with conjugated primary antibodies for 30 min on ice, washed twice in PBS/5%FCS, and fixed with the fixation buffer (eBioscience™ Foxp3/Transcription Factor Staining Buffer Set, Thermo Fisher Scientific) for 12 min at RT. For intracellular staining, after fixation, cells were permeabilized for 5 min at RT with the permeabilization buffer (eBioscience™ Foxp3/Transcription Factor Staining Buffer Set), then incubated with primary antibodies for 30 min at RT, washed twice in permeabilization buffer and finally resuspended in PBS/5%FCS until FACSing.

Antibodies used: mouse monoclonal antibodies anti-CD73 PE-conjugated (clone AD2), anti-CD90 FITC-conjugated (clone 5E10), anti-CD105 APC-conjugated (clone SN6), mouse IgG1 Kappa Isotype Control PE, FITC and APC (clone P3.6.2.8.1) and mouse IgM Isotype Control PE (eB121-15F9, all from eBioscience™, Thermo Fisher Scientific), anti-CD106 BV421-conjugated (clone STA), anti-CD146 BV711-conjugated (clone P1H12), anti-FSP1 FITC conjugated and mouse IgG1 Kappa Isotype Control BV421 and BV711 (clone MOPC-21, all from BioLegend), and anti-STRO-1 PE-conjugated (clone STRO-1, Proteintech).

### Cell growth

Cell growth was determined by seeding 10^5^cells/100 mm tissue culture dishes in their respective growth media. After attachment, cells were trypsinized and counted (*t* = 0) using a Neubauer counting chamber. Cultures were refed every other day and counted after 2, 5, and 7 days.

### In vitro differentiation and culture-specific staining

For in vitro differentiation, 10^5^cells were seeded into either 6-well plates (RNA extraction) or 35-mm tissue culture dishes (staining) in their regular growth medium and refed with lineage-specific medium 24 h afterwards.

*Osteogenesis* In vitro osteogenesis was induced by α-minimal essential medium (αMEM, Thermo Fisher Scientific) supplemented with 10%FCS, 1X PenStrep (Thermo Fisher Scientific), 0.05 mg/ml L-ascorbic acid, 0.01 M ß-glycerol phosphate and 0.1 mM dexamethasone (all from Sigma-Aldrich). Cultures were kept for 21 days, and medium exchanged every other day. At day 21, cells were fixed in 4% PFA, rinsed with ddH_2_O and incubated with Alizarin Red S (ARS, 68.45 mg Alizarin Red S (Sigma-Aldrich) in 5 ml ddH_2_O, pH 4.1–4.5) for 45 min at 4 °C in the dark. After extensive washing in ddH_2_O, samples were air-dried and observed with an Olympus BX-51 phase microscope (Olympus Life Science Solution). Images were analyzed with ImageJ. RNA was extracted from parallel cultures at days 7, 14, and 21.

*Adipogenesis* In vitro adipogenesis was induced by αMEM (Thermo Fisher Scientific) supplemented with 10% FCS, 1X PenStrep (Thermo Fisher Scientific), 0.5 mM 3-isobutyl-1-methylxanthine, 200 µM indomethacin, 10 µM insulin and 1 µm dexamethasone (all from Sigma-Aldrich). Cultures were kept for 14 days, and medium exchanged every other day. At day 14, cells were fixed in 4% PFA, rinsed with PBS and incubated with fresh Oil Red O solution (ORO; Stock solution (SS): 0.5 g Oil Red O (Sigma-Aldrich) in 100 ml isopropanol; Working Solution (WS): 6 ml SS in 4 ml ddH_2_O) for 1 h at RT. After extensive washing in PBS, samples were air-dried and observed with an Olympus BX-51 phase microscope. Images were analyzed with ImageJ. RNA was extracted from parallel cultures 3, 7 and 14 days after initiation.

*Chondrogenesis* In vitro chondrogenesis was induced by high-glucose DMEM (Thermo Fisher Scientific) supplemented with 2% PenStrep, 1% ITS^+^Premix (Thermo Fisher Scientific), 0.05 mg/ml L-ascorbic acid, 0.1 µM dexamethasone, 100 µg/ml sodium pyruvate, 40 µg/ml proline and 10 ng/ml TGFß3 (all from Sigma-Aldrich). Forty-eight hours after initiation, micro-mass formation was observed for all the cultures, which were kept for 21 days, exchanging medium every other day. At day 21, micro-masses were fixed overnight in 4% PFA at RT and processed for paraffin embedding and sectioning as described before. Sections were stained with Alcian Blue (AB, Sigma-Aldrich) and hematoxylin and observed with an Olympus BX-51 phase microscope. Micro-masses developed in parallel were used for RNA extraction at day 21.

### Colony-forming unit (CFU) assay

Ten thousand cells were seeded into p100 tissue culture dishes in their regular growth medium, refed every other day, and stained with CV at day 14. Colony number was assessed by using an automatized colony counter (aCOLyte, Synbiosis, Cambridge, UK). “Pooled Clones CLP” was gained from trypsinizing and pooling colonies formed after 14 days.

### Multiplex cytokine/chemokine array

To analyze the profile of secreted cytokines, 5 × 10^5^cells were seeded into p100 tissue culture dishes in their regular growth medium. Twenty-four hours afterwards, cells were washed twice in PBS and refed with fresh medium. CM was collected as described before. Five hundred microliters of CM was analyzed on a Human Cytokine Array/Chemokine Array 71-Plex Panel (HD71), (Eve Technologies, Calgary, AB, Canada). Parallel cultures were used for protein extractions allowing a normalization for the cytokine levels.

### Toll-like receptor priming

Lipopolysaccharide 1 µg/ml (LPS, Sigma-Aldrich) and poly(I:C) 100 µg/ml (Tocris Bioscience, Bristol, UK) were used as agonists for TLR4 and TLR3, respectively. In brief, cells were cultured to 70% of confluence in their growth medium, rinsed 2X in PBS and stimulated with LPS or poly(I:C) for 1 h. Cells were let to recover for 24 h prior to total RNA extraction.

### Single-cell clonal assay

To obtain single cell-derived clones, a limiting dilution method was used. In brief, cells were resuspended at a final concentration of 5 cells/ml and 100 µl of the cell solution was placed in each well of five 96-well plates. While this approach leads to approximately 50% of the wells being devoid of any cells, it ensured single cell-derived outgrowths as hardly any well contained more than one cell. In addition, cellular outgrowths were monitored daily, and each cell clone was individually expanded (clones were expanded from 96-well plates to 24-well plates, then to p35 and finally to p100 dishes). Single cell-derived clones were frozen and analyzed.

### Statistical analysis

Experiments were performed at least three times in multiple replicates. Data were analyzed using Prism 7 (GraphPad, La Jolla, CA, USA). Data are reported as means ± standard deviation (SD). Multiple comparisons were performed using one- or two-way analysis of variance (ANOVA) with Tukey’s post hoc test. Data were considered as significant when *p* < 0.05.

### Data availability

The datasets generated and/or analyzed during the current study are available from the corresponding author on reasonable request.

## Results

### CLP lip tissue is an optimal source of fibroblasts

**Fig. 1:**
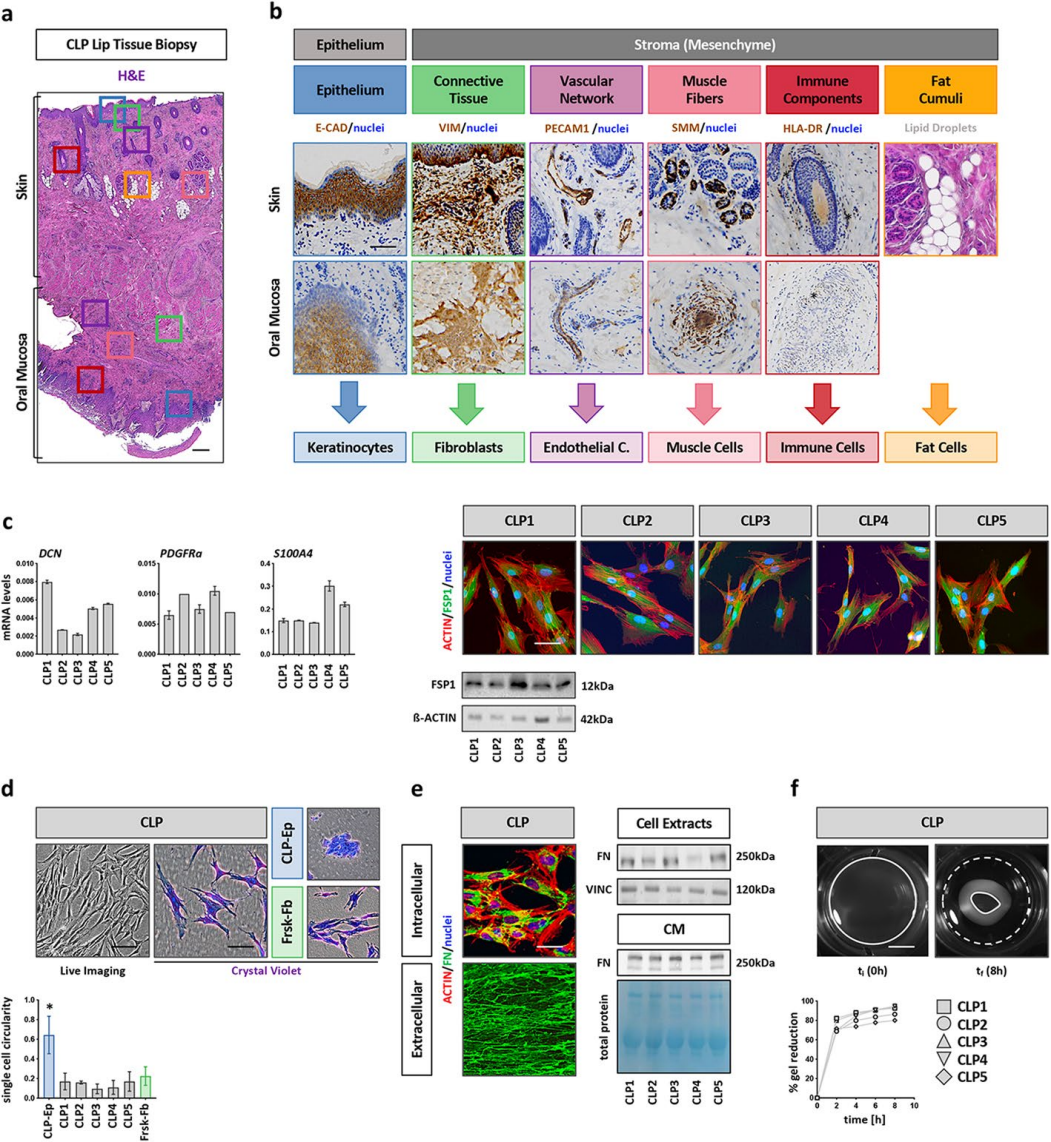
CLP tissue-derived fibroblasts characterization. **a** H&E staining of a representative CLP lip tissue biopsy shows the mucocutaneous transition between the skin (top) and the oral mucosa (bottom). Boxes indicate the main tissue structures that can be identified: epithelia (blue), connective tissue (green), vascular networks (purple), muscle fibers (pink), immune cells (red) and adipose cumuli (orange). Scale bar: 500 µm. **b** IHC of the CLP lip biopsy for E-CAD, VIM, PECAM1, SMM and HLA-DR prove the identity of the above-mentioned tissue-structures. Note that the fat droplets can be easily identified in the H&E section on the skin side of the lip. Each tissue component represents a potential source of primary cells as indicated on the bottom. Nuclei counterstain (blue) has been performed with hematoxylin. Stars indicate immune cells positive for HLA-DR. **c** qPCR analysis for *DCN*, *PDFGRa* and FSP1 (*S100A4*) in CLP1–CLP5. FSP1 expression was confirmed at protein level by immunoblot and IF (actin, red; FSP1, green; nuclei, blue). Full-length blots are presented in Additional file 1: Fig. S5. Scale bar: 20 µm. kDa: kilo Dalton. **d** Live and brightfield images after CV staining of CLP, CLP-Ep (negative control), and Frsk-Fb (positive control). Scale bars: 100 µm (Live Imaging); 50 µM (CV). The graph shows the cc for 50 single cells. *p < 0.05 CLP-Ep versus CLP1–CLP5 or Frsk-Fb. CLP-Ep: CLP-derived epithelial cells, Frsk-Fb: foreskin-derived fibroblasts. **e** IF staining for intracellular (top) and extracellularly (bottom) deposited fibronectin (FN, green). Scale bar: 20 µm. Actin (red); nuclei (blue). Intracellular (total cell extracts) and secreted (conditioned medium) FN was also analyzed by immunoblot. Full-length blots are presented in Additional file 1: Fig. S5. kDa: kilo Dalton. **c** Live imaging pictures of collagen gels seeded with CLP before (left) and after contraction (right). White dashed lines indicate the initial area of the gels, while the white lines indicate the final size of the gel. Scale bar: 7.5 mm. Progressive gels reduction was measured by ImageJ over a period of 8 h and plotted in the diagram on the bottom. t_i_: initial time point; t_f_: final time point

The surgical correction of the cleft lip at the infant’s age of 3–6 months requires the precise excision of superfluous tissue reaching into the cleft (Additional file 1: Fig. S1a). This tissue, which is usually discarded, represents a source for the isolation of various primary cells. A representative H&E-stained section of such biopsies shows the mucocutaneous junction of lip tissue, where the skin (top) transitions into oral mucosa (bottom) (Fig. 1a). All anatomical structures that might result in potential cell isolations were assessed by IHC (Fig. 1b): Stratified epithelia (keratinocytes) were stained for E-Cadherin (E-CAD, blue box), connective tissue (stromal cells) for vimentin (VIM, green box), vascular networks (endothelial cells) for platelet endothelial cell adhesion molecule-1 (PECAM1, purple box), smooth muscle cells lining the walls of blood vessels for smooth muscle myosin (SMM, pink box), and immune cells for HLA-DR (red box). Fat tissue (adipocytes) could be identified by the presence of lipid droplet accumulations (orange box) in the H&E picture.

From five randomly chosen CLP lip tissue donors, we isolated and expanded two morphologically distinct cell outgrowths (Additional file 1: Fig. S1b): (1) cobblestone-like cells and (2) elongated and scattered cells. To determine the origin of these cells, we co-stained the cultures for E-CAD and VIM. While the densely packed cells were positive for E-CAD, VIM was only expressed by the spindle-like cells, suggestive of epithelial and connective tissue origin, respectively. To further assess the exact cellular identity and to determine the purity of our isolated primary stromal cells, which we call CLP1–CLP5 (see Table 1), we analyzed them for their expression of the cell type-specific genes (see above) and compared them to respective reference cell lines (Additional file 1: Fig. S1c). CLP1–CLP5 displayed significantly lower levels of *CDH1*, *PECAM1*, *MYH11*, *HLA-DR*, and *SLC7A10* (marker for adipocytes) compared to corresponding controls. In contrast, *VIM* transcripts were similarly detectable in CLP1–CLP5 and a foreskin fibroblast reference cell strain (Frsk-Fb). Although VIM is recognized as a marker of fibroblasts, it is known that additional mesenchymal cells also express it (e.g., endothelial, muscle or immune cells, data not shown). Hence, we further confirmed fibroblast identity of CLP1–CLP5 cultures by assessing levels of additional genes and proteins enriched in fibroblasts (Fig. 1c). Decorin (*DCN*), platelet-derived growth factor receptor-*a* (*PDGFRa*), and fibroblast-specific protein 1 (FSP1, gene: *S100A4*) were all robustly expressed in CLP1–CLP5. We validated FSP1 protein expression in CLP1–CLP5 by immunoblotting and IF staining. All these data suggest that our explant system allows us to isolate fibroblasts from discarded CLP lip tissue without any contaminations from epithelial, endothelial, muscle, immune, or fat cells.

Next to screening our CLP1–CLP5 cultures for the presence of fibroblast markers, we also wanted to assess the presence of fibroblast-specific morphological and functional traits (i.e., ECM production and generation of contractile forces). Live imaging and CV staining of CLP1–CLP5 revealed a typical spindle-like cell morphology, which was identical to Frsk-Fb, but in stark contrast to epithelial cells (CLP-Ep) (Fig. 1d). This visual observation was confirmed quantitatively by determining the cell circularity (cc) within the cultures, which showed that CLP1–CLP5 and Frsk-Fb are more elongated with a lower cc index than CLP-Ep. Afterwards, we evaluated the potential of CLP1–CLP5 to synthesize, secrete, and deposit the ECM molecule fibronectin (FN) (Fig. 1e). Using IF and immunoblots, we demonstrate that FN is synthesized (intracellular staining and total cell extracts), secreted (CM), and deposited (staining of extracellular decellularized matrix) by CLP1–CLP5. Finally, we performed a collagen contraction assay to assess the capacity of our CLP lip fibroblasts to generate contractile forces (Fig. 1f). CLP1–CLP5 were able to significantly contract a collagen gel within 8 h. Availability of all these properties confirmed fibroblast identity of CLP1–CLP5.

### CLP lip fibroblasts possess MSC-like traits

**Fig. 2:**
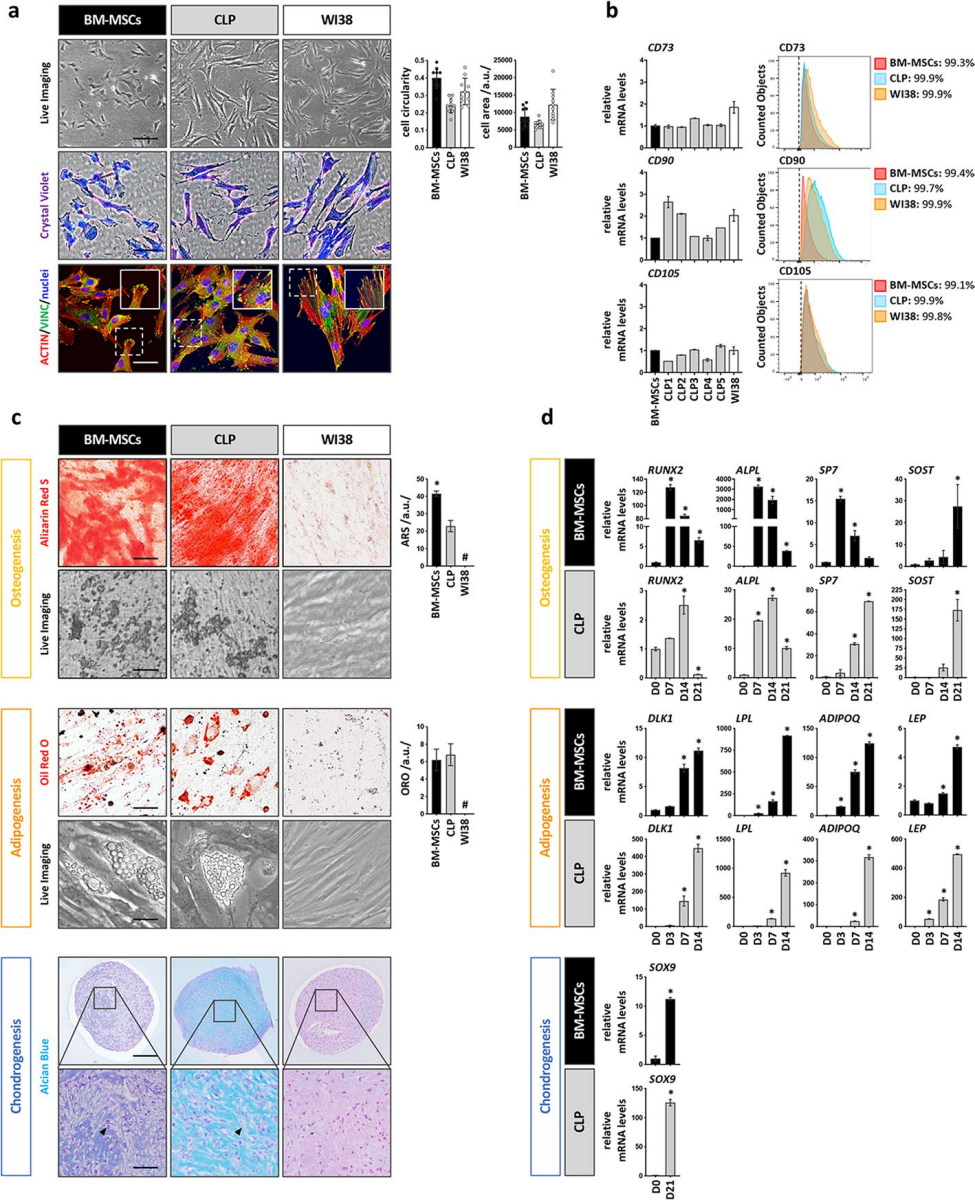
Evaluation of the 3 main MSCs-like properties in CLP lip fibroblasts. **a** Live, brightfield, and IF images of BM-MSCs, a CLP lip fibroblast culture and WI38 lung fibroblasts cell line. Scale bars: 100 µm (Live Imaging), 50 µm (CV), 20 µm (IF). Vinculin (VINC, green); actin (red); nuclei (blue). Histograms to the right report the cc and cell areas of the three cell populations analyzed in 50 individual cells. a.u.: arbitrary unit. **b** qPCR (left) and FACS analysis (right) for the MSCs surface markers CD73, CD90, and CD105 in BM-MSCs (set to 1), CLP1–CLP5 (FACS: CLP1 only is shown) and WI38. Black dashed lines in the FACS plots indicate the threshold of unstained samples. The percentage of positive cells for each sample is reported. Gating strategy is presented in Additional file 1: Fig. S6. **c** At the end of the differentiation time mineralization (yellow box), lipid droplet accumulation (orange box) and presence of cartilage-related ECM components (blue box) in BM-MSCs, a CLP culture, and WI38 were assessed by ARS, ORO and AB staining, respectively. For osteogenesis and adipogenesis live imaging pictures are also shown (day 21 and day 14, respectively). Quantifications of ARS and ORO by ImageJ are reported on the right. Arrows in the AB pictures indicate hypertrophic cells. Scale bars: 5 µm (ARS, ORO and Live Imaging); 200 µm (AB); 20 µm (AB close-ups). *p < 0.05 BM-MSCs versus CLP. # no detected staining. a.u.: arbitrary unit. **d** qPCR analysis of osteogenic (*RUNX2*, *ALPL*, *SP7*, and *SOST*), adipogenic (*DLK1*, *LPL*, *ADIPOQ* and *LEP*) and chondrogenic-associated (*SOX9*) genes in BM-MSCs and a CLP culture at different timepoints during differentiation. The expression of each marker has been normalized to its expression at day 0 (D0). *p < 0.05

In the past, fibroblasts have often been used as negative controls for the evaluation of MSC-like properties [42]. However, more recent evidence is emerging that fibroblasts also share several hallmarks with MSCs [21, 30, 31]. Therefore, we wanted to elucidate similarities and differences between CLP1–CLP5, BM-MSCs (positive control) and WI38 cells, an established fibroblast cell line, which is known to lack any multilineage potential (negative control) [32] regarding the three minimal MSC-defining criteria [14]. Live imaging and CV showed that CLP1–CLP5 cultures are composed of spindle-like and plastic-adherent cells having similar cellular cell size and cc to BM-MSCs and WI38. Also, IF staining for actin and the focal adhesion-associated protein vinculin did not reveal any obvious differences among the various cultures (Fig. 2a). Similarly, expression of the MSC-related surface markers CD73, CD90, and CD105 was identical between the three cell groups with more than 95% of the cells being positive for all three proteins (Fig. 2b). In addition, none of the cell cultures significantly expressed the hematopoietic markers *CD31* and *CD45* (Additional file 1: Fig. S2a) and all displayed similar proliferation rates (Additional file 1: Fig. S2b). We next evaluated the trilineage differentiation capacity of three CLP strains (CLP1–CLP3) compared to BM-MSCs and WI38. In contrast to WI38, CLP1–CLP3 and BM-MSCs could be committed into bone-, fat- and cartilage-forming cells as assessed by live imaging pictures and lineage-specific stains after differentiation (Fig. 2c). Quantification of the ARS staining revealed an increased signal in BM-MSCs compared to CLP1–CLP3, while ORO levels were similar between CLP1–CLP3 and BM-MSCs. Successful chondrogenesis was indicated by a positive AB staining as well as by the presence of dispersed and cytoplasm-rich cells in CLP1–CLP3- and BM-MSC-derived micro masses. These properties are reminiscent of hypertrophic chondrocytes during endochondral ossification [43]. Next, we aimed to comparatively assess the induction of several lineage-specific differentiation markers in CLP1–CLP3 and BM-MSCs (Fig. 2d). Osteogenic commitment of BM-MSCs was associated with an earlier peak of induction for the genes *RUNX2, ALPL*, and *SP7* (day 7 in BM-MSCs versus day 14/21 in CLP1–CLP3)*,* and a higher elevation of *RUNX2* and *ALPL* when compared to CLP1–CLP3. In contrast, the osteocyte marker *SOST* peaked at day 21 of differentiation in both CLP1–CLP3 and BM-MSCs, while its fold induction was significantly higher in CLP1–CLP3 compared to BM-MSCs. While these molecular data might explain the increased ARS signal in BM-MSCs versus CLP1–CLP3, it should be noted that the expression of the osteogenic markers was also highly variable between the two groups at basal conditions (Additional file 1: Fig. S2c). Gene expression analyses along the adipogenic and chondrogenic commitment did not exhibit any significant difference in the temporal inductions of the genes between BM-MSCs and CLP1–CLP3. However, the elevations of *DLK1* and *LEP* (adipogenesis) and *SOX9* (chondrogenesis) were more pronounced in CLP1–CLP3 than in BM-MSCs.

### CLP lip fibroblasts possess additional MSC-like properties

**Fig. 3:**
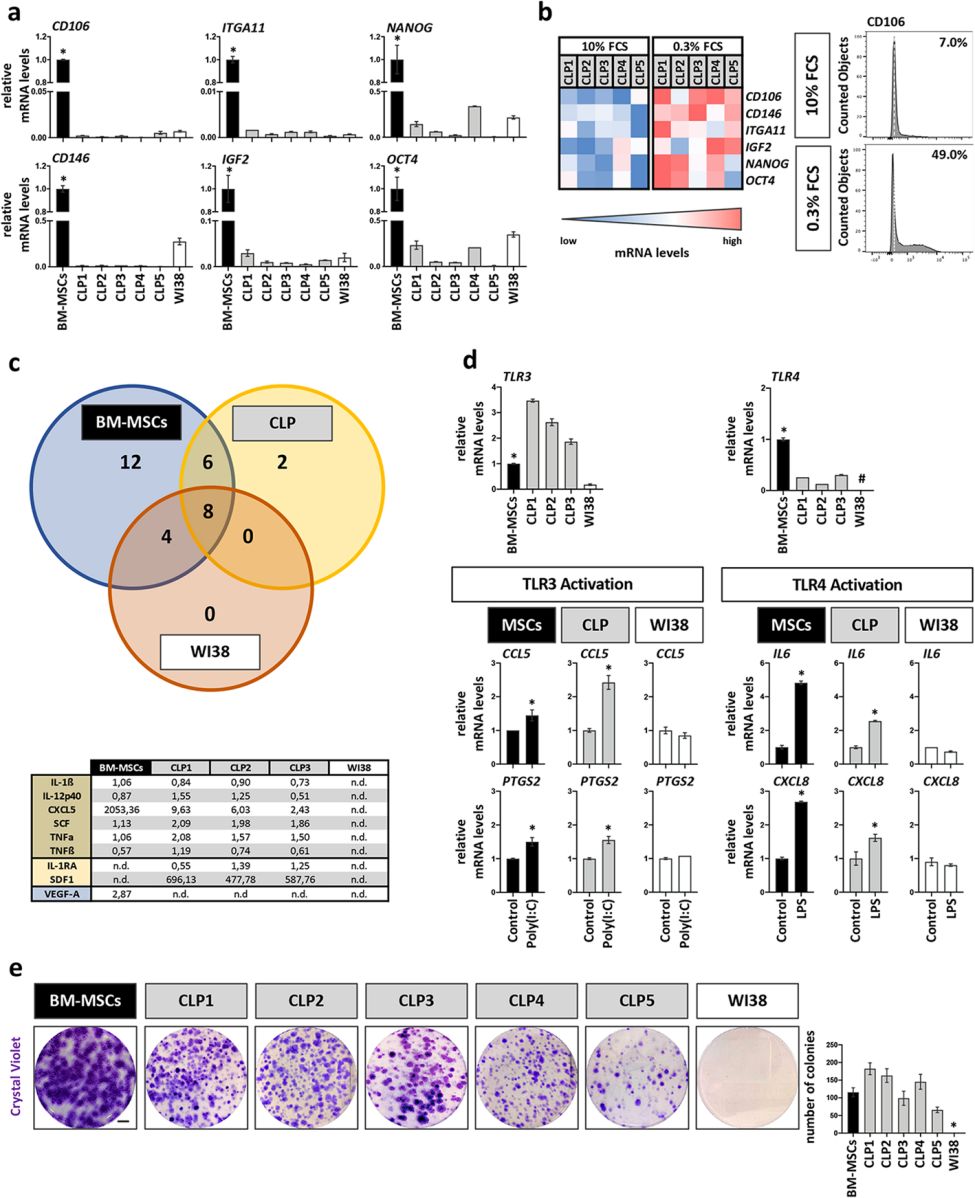
Evaluation of additional MSC-like properties in CLP lip fibroblasts. **a** qPCR analysis for the expression of newer MSC markers *CD106*, *CD146*, *ITGA11*, and *IGF2*, and of the embryonic pluripotency markers *NANOG* and *OCT4* in BM-MSCs (set to 1), CLP1–CLP5 and WI38 cells. *p < 0.05 BM-MSCs versus CLP1–CLP5 or WI38. **b** The same set of genes was analyzed in CLP1–CLP5 under starving (0.3% FCS, 24 h) and regular (10% FCS) culturing conditions by qPCR. Absolute mRNA levels are shown in the heatmap (blue, low expression; red, high expression). Note that starvation induces the expression levels of these genes in all CLP cultures. Increased CD106^+^ population under low serum conditions was confirmed by FACS. White dashed lines indicate the unstained sample threshold. The percentage of positive cells is reported. Gating strategy is presented in Additional file 1: Fig. S6. **c** Venn diagram comparing the secretion of 32 different pro- and anti-inflammatory cytokines in the conditioned medium of BM-MSCs, a CLP culture, and WI38 cells. In the table quantification of the cytokines common to BM-MSCs and CLP or exclusive for CLP and BM-MSCs in case of VEGF-A is reported. Quantification data should be read as pg/ml. n.d. no determined. **d** Assessment of *TLR3* and *TLR4* expression in BM-MSCs, CLP1–CLP3 and WI38 by qPCR. The expression levels were normalized to BM-MSCs. *p < 0.05 BM-MSCs versus CLP1–CLP3 or WI38. # no detected gene (Ct value > 32). TLR3- and TLR4-mediated target gene expression in BM-MSCs, a CLP culture, and WI38 was analyzed by qPCR for *CCL5* and *PTGS2*, and of *IL6* and *CXCL8* after activation of TLR3 by poly(I:C) and TLR4 by LPS, respectively. Note that WI38 cell line is not responsive to TLRs stimulation. The expression levels under activating conditions were normalized for each sample to their relative basal expression. *p < 0.05. **e** Pictures as well as quantification of single cell-derived colonies of BM-MSCs, CLP1–CLP5, and WI38 stained with CV after 14 days. Scale bar: 1 cm. *p < 0.05 WI38 versus BM-MSCs or CLP1–CLP5

By now it seems clear that CD73, CD90, and CD105 are not ideal markers for the discrimination of MSCs from other cell types not having multipotent characteristics (i.e., WI38, see Fig. 2c and [32]). Hence, we wished to address expression levels of other reported MSC-related markers, such as STRO-1, CD106, CD146, ITGA11, and IGF2 [44–46], as well as of markers specific for the maintenance of the pluripotent state in embryonic stem cells (*NANOG* and *OCT4*) in CLP1–CLP5. All these markers were detectable in CLP1–CLP5 (Fig. 3a), although at a significantly lower level than in BM-MSCs, and except for STRO-1, which was not present in BM-MSCs (Additional file 1: Fig. S3a). The fact that their expression in CLP1–CLP5 was comparable (e.g., CD106) or lower (e.g., STRO-1, CD146) to their levels in WI38 questions their use as specific markers for the identification of cells with multipotent capacities in vitro. To partially justify their suitability as MSC markers, we checked whether their levels could be modulated by harsh culturing conditions. Indeed, it is believed that MSC properties as well as levels of MSC markers can be affected by local cues derived from their unique microenvironment (e.g., ECM stiffness, hypoxia, nutrient deprivation) [24, 47]. Accordingly, MSCs should survive better than other cells in harsh conditions [48]. Mimicking harsh conditions, by culturing CLP1–CLP5 under serum deprivation (0.3% FCS), elicited a marked induction of *CD106*, *CD146*, *ITGA11*, and *IGF2*, but also of the pluripotency markers *NANOG* and *OCT4* (Fig. 3b), but not of *CD73*, *CD90*. and *CD105* (Additional file 1: Fig. S3b) when compared to regular culturing conditions (10% FCS). This observation was specific for CLP1–CLP5, as BM-MSCs and WI38 did not respond to reduced serum levels (Additional file 1: Fig. S3c). We confirmed some of these results on protein level and determined an increase of the CD106^+^ population from 7 to 49% in CLP.

MSCs are appealing for clinical use because next to their multilineage differentiation ability, they also possess additional traits, such as the capacity to secrete cytokines and modulate the response of immune cells, as well as to have a high proliferative efficiency [15–17]. Therefore, we were keen to learn whether our CLP1–CLP5 also shares these properties with BM-MSCs.

First, we profiled the CMs of CLP1–CLP3 for the presence of pro- and anti-inflammatory cytokines and compared them to BM-MSCs and WI38 (Fig. 3c, Additional file 1: Fig. S3d and Additional file 1: Table S2). A total of 16 cytokines were identified to be constitutively secreted by CLP1–CLP3, while 30 and 12 were detected in BM-MSCs and WI38 CMs, respectively. Eight cytokines were common to all the three cell groups, including interleukin-6 (IL6), whose levels were the highest in CLP1–-CLP3. Six cytokines (interleukin-1ß IL-1ß, interleukin-12 ß subunit IL-12p40, chemokine 5 C-X-C motif CXCL5, stem cell factor SCF and tumor necrosis factors *a* TNF*a* and ß TNFß) were only shared between BM-MSCs and CLPs, while two cytokines, stromal cell-derived factor-1 (SDF-1) and the interleukin-1 receptor antagonist (IL-1RA), were exclusively detected in CLP1–CLP3. Among the BM-MSC-specific cytokines, VEGF-A was detected, which suggests an increased pro-angiogenic capacity of BM-MSCs compared to CLPs and WI38.

Next, we wished to investigate whether CLP lip-fibroblasts have an immunomodulatory capacity as it has been described for MSCs (Fig. 3d). To exert their immunomodulatory activities, MSCs require to be “primed” by inflammatory mediators present in the microenvironment through Toll-like receptors (TLRs) 3 and 4 activation [49]. Both *TLR3* and *TLR4* transcripts were detectable in CLP1–CLP3. While *TLR3* levels were higher in CLP1–CLP3, *TLR4* were lower when compared to BM-MSCs. In contrast, WI38 expressed only minimal levels of *TLR3*, but were devoid of *TLR4*. In order to measure the release of TLR3-dependent inflammatory mediators C–C motif chemokine ligand 5 (*CCL5*), prostaglandin-endoperoxide synthase 2 (*PTGS2*) as well as of TLR4-specific interleukin-8 (*CXCL8*) and *IL6*, we pharmacologically activated the two receptors [50]. Activation of TLR3 by Poly(I:C) in CLP1–CLP3 and BM-MSCs significantly induced the levels of *CCL5* and *PTGS2*, whereas LPS stimulation of TLR4 in the same cells resulted in an elevation of *IL6* and *CXCL8* when compared to control conditions. As expected, there was no increased cytokine release in WI38 upon treatment, which fits to their neglectable levels of *TLR3* and *TLR4*.

Lastly, we assessed the proliferative efficiency of the CLP1–CLP5 by performing a colony-forming unit assay (CFU) (Fig. 3e). Similar to BM-MSCs, but in contrast to WI38, CLP1–CLP5 were prominently able to form colonies derived from single cells. However, the CLP-derived colonies were slightly smaller and less dense compared to the ones formed by BM-MSCs.

### CFU assay does not enrich for CLP lip fibroblasts with improved MSC-like traits

**Fig. 4:**
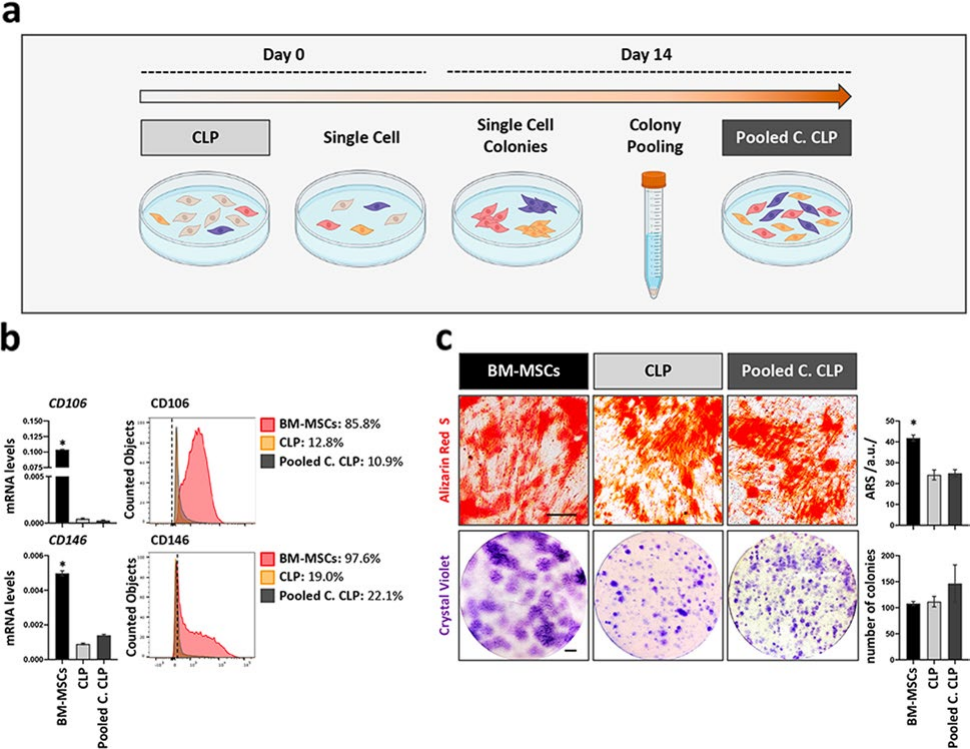
Evaluation of the MSC-like properties in the Pooled Clones CLP lip fibroblasts. **a** Diagram of the strategy used to obtain the Pooled Clones CLP (Pooled C. CLP) population. **b** qPCR (left) and FACS (right) analysis for the expression of the MSC-surface markers CD106 and CD146 in BM-MSCs, the parental CLP and the Pooled Clones CLP population. Black dashed lines in the FACS plots indicate the threshold of unstained samples. The percentage of positive cells for each sample is reported. Gating strategy is presented in Additional file 1: Fig. S6. *p < 0.05 BM-MSCs versus CLP or Pooled Clones CLP. **c** Brightfield images after ARS (day 21) and CV staining (day 14) show, respectively, mineralization and single-cell derived colonies in BM-MSCs, parental CLP and Pooled Clones CLP populations. Histograms to the right report quantification of the ARS staining by ImageJ and quantification of the colonies. Scale bars: 5 µm (ARS); 1 cm (CV). *p < 0.05 BM-MSCs versus CLP or Pooled Clones CLP. a.u.: arbitrary unit

The possibility to modulate the expression of *CD106*, *CD146*, *ITGA11*, *IGF2*, *NANOG* and *OCT4,* as well as the fact that only a fraction of cells expresses them, let us believe that the CLP1–CLP5 populations are heterogenous. This challenged us to analyze our CLP cultures for the presence of subpopulations with enhanced MSC-like traits. Therefore, we explored the possibility to enrich for a population with higher MSC marker expression and increased MSC-like properties by pooling cells that were able to form single cell-derived colonies indicative of a more efficient proliferative capacity typical for MSCs (“Pooled Clones CLP”) (Fig. 4a). However, “Pooled Clones CLP” still expressed equal and significantly lower levels of the two novel and mostly described MSC-related markers, CD106 and CD146, when compared to the parental CLP and BM-MSCs, respectively (Fig. 4b). Similarly, the osteogenic as well as the CFU potential was not enhanced in the “Pooled Clones CLP” versus parental CLP (Fig. 4c). These data imply that either there are no subpopulations with superior MSC-like activities within our CLP cultures or that the CFU assay is not a suitable method for the selection of cells with increased multipotent characteristics.

### Single cell-derived clones display variable MSC-like properties but do not have superior capacity compared to the parental CLP population and are still fibroblasts

**Fig. 5:**
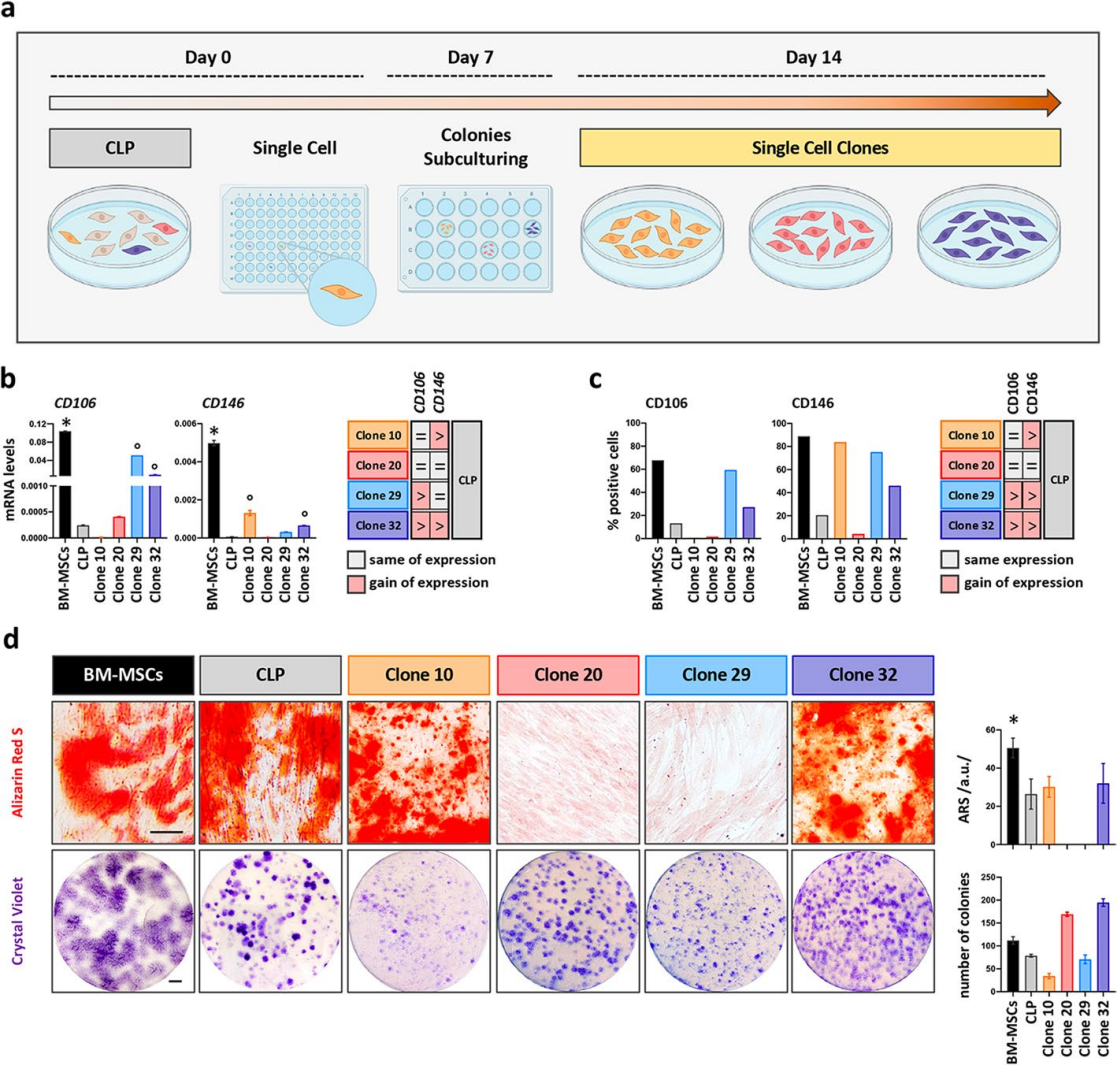
Evaluation of the MSC-like properties in CLP single cell-derived clones. **a** Diagram of the strategy used to obtain single cell clones. **b** qPCR analysis for the expression of the MSC-surface markers *CD106* and *CD146* in BM-MSCs, the parental CLP and 4 representative clones (10, 20, 29 and 32). In the table to the right comparable or gained expression of these markers in the clones when compared to CLP is reported. *p < 0.05 BM-MSCs versus CLP and clones; °*p* < 0.05 CLP versus clones. **c** qPCR data were similarly confirmed by FACS analysis of CD106 and CD146 in the same groups. Gating strategy and histograms are presented in Additional file 1: Fig. S6. *p < 0.05 BM-MSCs versus CLP and clones; °*p* < 0.05 CLP versus clones. **d** Brightfield images after ARS (day 21) and CV staining (day 14) show mineralization and single-cell derived colonies in BM-MSCs, CLP and Clones 10, 20, 29 and 32. Histograms to the right report quantification of the ARS staining by ImageJ and quantification of the colonies. Scale bars: 5 µm (ARS); 1 cm (CV). *p < 0.05. a.u.: arbitrary unit

Finally, we decided to perform a single cell clonal analysis to decipher whether we could select for clones having superior MSC-like characteristics (Fig. 5a). Thirty single cell-derived clones were screened for *CD106* and *CD146* expression by qPCR (Additional file 1: Fig. S4a), which revealed retention of the significantly lower levels of the markers in all clones when compared to BM-MSCs. Still, the levels of CD106 and/or CD146 were modulated in the majority of the clones compared to parental CLP: Five clones (17%) gained *CD146* expression while showing unchanged *CD106* levels, 15 clones (50%) gained *CD106* with unaltered levels of *CD146*, and six clones (20%) gained the expression of both markers. Only four clones (13%) displayed similar *CD106* and *CD146* levels to the parental CLP. Based on these findings, we selected four specific cell clones (Clones 10, 20, 29, and 32) with variable *CD106/CD146* levels for further and more in-depth analyses. We initially wished to confirm their *CD106* and *CD146* mRNA levels (Fig. 5b) at protein level by FACS (Fig. 5c and Additional file 1: Fig. S6e), which was the case for three clones, but not for Clone 29. Clone 29 displayed increased CD146 protein when compared to parental CLP, which did not match with the initial mRNA assessment. Nevertheless, we kept this clone in our analysis. Additionally, we further assessed the expression of STRO-1 in the selected clones (Additional file 1: Fig. S4c). Compared to the parental CLP population, we observed a considerable expansion of the STRO-1^+^ population in Clone 10 and a slight increase in Clone 29 and 32, while unchanged levels were detected for Clone 20. The selected clones were then evaluated for their osteogenic and CFU potential by comparing them to the parental CLP strain and to BM-MSCs (Fig. 5d). While the CFU capacity was maintained by all the clones, although with variable efficiency, osteogenesis was only identified in clones 10 and 32, but not in clones 20 and 29 by ARS staining. However, we could not determine an increased osteogenic potential in the clones 10 and 32 when compared to parental CLP strain.

**Fig. 6:**
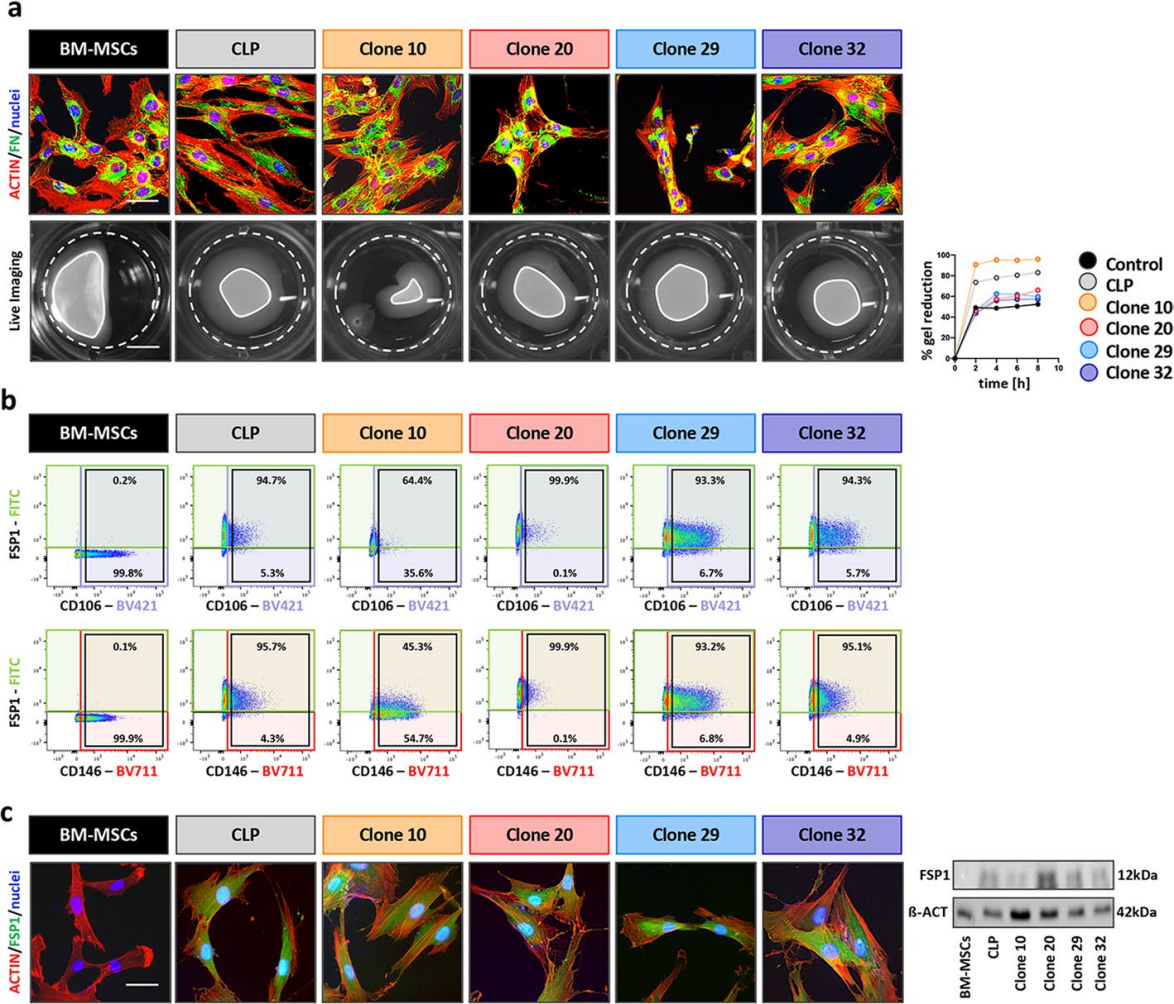
**a** IF images of BM-MSCs, CLP and Clones 10, 20, 29 and 32 for human FN (green). Scale bar: 20 µm. Actin (red); nuclei (blue). Live imaging pictures of detached collagen gels seeded with BM-MSCs, CLP and Clones 10, 20, 29 and 32 (time:8 h). White dashed lines indicate the initial area of the gels, while the white lines indicate the final size of the gels. Scale bar: 7.5 mm. Progressive gels reduction was measured by ImageJ over a period of 8 h and plotted in the diagram on the right. **b** Scatter plots display the co-expression of CD106 and FSP1 (top row), or of CD146 and FSP1 (bottom row) in BM-MSCs, CLP and Clones 10, 20, 29 and 32. CD106, CD146 and FSP1 positive cells are included in the purple, red and green boxes, respectively. Black boxes indicate CD106 or CD146 populations. Percentages indicate the fraction of CD106 or CD146 positive cells that are positive (top) or negative (bottom) for FSP1 expression. Gating strategy is presented in Additional file 1: Fig. S6. **c** IF staining and immunoblot for FSP1 protein expression confirmation in BM-MSCs, CLP and Clones 10, 20, 29 and 32. Full-length blots are presented in Additional file 1: Fig. S5. Scale bar: 20 µm. FSP1 (green); actin (red); nuclei (blue). kDa: kilo Dalton

Prompted by the observation that a major fraction (26/30, 87%) of the cell clones gained expression of at least one of the tested MSC markers, we wanted to know whether these cells lost some of their fibroblast characteristics (Fig. 6a). The levels of the ECM protein FN were comparable in BM-MSCs, parental CLP and the four single cell-derived clones analyzed. However, the capacity of the clones to generate contractile forces was as efficient as the parental CLP strain and significantly more than BM-MSCs. In addition, we analyzed the co-expression of the MSC markers CD106 and CD146 with the fibroblast marker FSP1 by FACS (Fig. 6b). BM-MSCs were found to be negative for FSP1. However, both the parental CLP population and the four clones showed robust FSP1 levels. We also observed that the majority of FSP1^+^ CLP cells also co-expressed the MSC-associated markers CD106 and CD146. These observations suggest that FSP1 might be a marker being able to discriminate fibroblasts from MSCs, while questioning the use of CD106 and CD146 as MSC-specific molecules in vitro. FSP1 absence in BM-MSCs and its presence in CLP lip-derived fibroblasts was further shown by IF staining and immunoblot (Fig. 6c). To conclude, our data provide strong evidence that MSC-like properties are present in heterogeneous CLP lip fibroblasts and that selection of a single subpopulation within our CLP culture does not improve the osteogenic differentiation potential.

## Discussion

The lip is composed of skin and oral mucosa. Within the stroma of these two compartments, different cell types exist, including fibroblasts, which are known to be the most abundant cells, endothelial, muscle, immune, and fat cells, but also multipotent cells [51, 52]. From CLP lip biopsies, we isolate spindle-shaped cells using the explant system and culture these cells in basic growth medium containing 10%FCS. With this approach, we already select for robust, readily available, and FCS-tolerating cells, since other more delicate cell types (e.g., myoblasts, endothelial cells) require more specialized culturing conditions [53–56]. We morphologically, genetically, and functionally identified these cells as fibroblasts (Fig. 1). Although fibroblasts have long been thought to be terminally differentiated, different studies have shown that dermal skin-derived fibroblasts share a MSC phenotype and possess a multilineage differentiation potential similar to MSCs [57–59]. Herein, we confirmed these and more recent studies [60–62] and showed that our CLP lip-derived fibroblasts (CLP1–CLP5) fulfill the minimal MSC-defining criteria [14, 17]. Indeed, CLP lip-derived fibroblasts and MSCs are barely distinguishable cells regarding their morphology, expression of specific cell surface markers (CD73, CD90, CD105), and trilineage differentiation potential (Fig. 2) [21, 30–33]. However, the analysis of cell differentiation at a molecular level revealed a certain difference between CLPs and BM-MSCs. While in CLP lip-derived fibroblasts a greater induction of the fat- and cartilage-related genes was determined, elevation of bone-related genes was diminished compared to BM-MSCs. The differences in the bone- and cartilage-related genes can be explained by their variable basal levels in BM-MSCs and CLP1–CLP3 (Additional file 1: Fig. S2c). On the other hand, a stronger induction of fat-associated genes might reflect the distinct tissue source of CLP cells and BM-MSCs [63]. In addition, we extended the characterization of CLP lip-derived fibroblasts and showed that CLP1–CLP5 express additional MSC markers and have the capacity to form colonies from single cells and to modulate immune responses (Fig. 3). To the best of our knowledge, these findings have never been reported by others for fibroblast cultures. The high similarity between CLP lip-derived fibroblasts and BM-MSCs might arise from a small MSC population being co-present in our CLP population. However, it is doubtful that such MSCs would survive long-term in regular culture medium as they are significantly less abundant than fibroblasts and require specific culturing media [19, 20]. A single cell clonal analysis further excluded this possibility (Figs. 5, 6). Indeed, single cell-derived clones retained the expression of the fibroblast marker FSP1, which proves that even the single cell-derived clones are still composed of fibroblasts. Notably, we report FSP1 as the best marker being able to distinguish BM-MSCs from CLP lip-derived fibroblasts. While fibroblasts robustly expressed the gene and the protein, FSP1 was not detectable in BM-MSCs, which has not been described so far. Of course, we cannot exclude the possibility that the difference in FSP1 expression might be caused by the fact that we compared neural crest-derived cells (CLP) from mostly mesoderm-derived BM-MSCs, although there is evidence that BM-MSCs also contain cells from the neural crest [64, 65]. Our study also raises the question about the nature and identity of fibroblasts and/or MSCs and their relation to each other, as the proposed criteria for defining MSCs [14, 17] are not sufficient to discriminate the two populations.

The easy accessibility of fibroblasts with multipotent capabilities from regularly discarded lip tissue is intriguing for several reasons.

(1) The lip is formed by fusion processes involving several facial prominences that are mostly derived from neural crest cells (NCCs) [66]. NCCs, which might persist in adult tissues [67], are known to display a strong multipotent character during embryogenesis as they give rise to various structures, including facial derivatives [68]. Whether our neural crest-derived CLP lip-derived fibroblasts were imprinted with a strong multipotent character by their embryonic origin remains speculative at this point. However, similar findings were observed in a recent study that showed a better performance of neuroectoderm-derived nasal chondrocytes than mesoderm-derived articular chondrocytes in the generation of functional cartilaginous tissue [69]. Although no one has yet tested intraindividual fibroblasts derived from different germ layers for their multi-plastic behavior, CLP lip-derived fibroblasts might benefit from their embryological origin. The comparison of patient-matching fibroblasts isolated from tissues of different embryological derivations will be one of our future aims.

(2) We isolate fibroblasts from very young tissue donors (3–6-month-old infants). It is known that the donor age greatly reduces the proliferative efficiency of BM-MSCs, while it accelerates apoptosis and senescence [70, 71]. In fact, there seems to be a general drop in quantity and quality of BM-MSCs with age, which might impair their clinical use when isolated from older patients [72]. A possible explanation for the reduced potential of MSCs isolated from old donors could be associated with the downregulation of transcription factors required for maintaining self-renewal and multipotency of embryonic stem cells (e.g., *NANOG* and *OCT4*) [73]. In this regard, it has been described that NANOG overexpression in adult-derived BM-MSCs rejuvenates their proliferation and differentiation potential to a similar extent to neonatal BM-MSCs [74]. Another study showed that in vitro passaging of MSCs (i.e., in vitro aging) downregulated *NANOG* and *OCT4* with consequent impairment of their multipotent potential. On the contrary, enrichment of *NANOG* and *OCT4*-positive MSC populations, which can be achieved by harsh culturing conditions, promotes their stemness [24]. A similar response could be observed in our study upon serum deprivation of CLP1–CLP5. Starvation resulted in an increase of *NANOG* and *OCT4*, but also of the more recently described MSC-related markers (*CD106*, *CD146, ITGA11*, and *IGF2*, Fig. 3b). These data suggest that our CLP lip-derived fibroblast behave comparable to BM-MSCs regarding their response to external cues. As such, the expression of *NANOG* and *OCT4* in our CLP lip-derived fibroblasts, which can be tunable by environmental cues, let us believe that the young age of our CLP tissue donors may be beneficial.

(3) Our CLP lip-derived fibroblasts are heterogeneous populations. Although fibroblast heterogeneity might be initially conceived as a hurdle since it might require a selection for a more potent subpopulation for clinical applications, our results convincingly show the opposite: no single subpopulation possessed superior multipotent characteristics when compared to the parental CLP strain. Hence, there is no need for in vitro selection and expansion of specific cell clones. Heterogeneity in our populations might be derived from the complex anatomy of the lip tissue, but also from the concept that not all fibroblasts are equal within tissues. In accordance with this, recent findings have revealed the existence of at least four different fibroblast clusters within human skin and oral mucosa dermis in vivo: (1) secretory-reticular, (2) secretory-papillary, (3) pro-inflammatory and (4) mesenchymal fibroblasts [37, 38]. Each subpopulation fulfills distinct fibroblast functions and can be identified by specific markers. Noteworthy, the mesenchymal cluster, which annotates fibroblasts with differentiating potential, was found to be reduced in old donors [37], which further reinforces the benefit of our young donors as described above. CLP lip-derived fibroblasts expressed most of the representative markers defining each cluster (data not shown). This finding indicates that all four fibroblast clusters are present in our cultures, which contributes to their heterogeneity. Some of these fibroblast clusters anatomically co-localize [37] making their purification impossible. To bypass this problem, we performed a clonal analysis. Single cell-derived clones were gained expressing variable CD106, CD146, and STRO-1 levels and possessing different potentials for osteogenesis and CFU. However, none of the clones displayed a superior MSC-like capacity when compared to the parental CLP population (Fig. 5). In a heterogeneous population as found in our CLP lip-derived fibroblasts, the success of the whole culture might depend on the contribution of each single cell. Even presence of fibroblasts without certain MSC-like traits (e.g., lack of bone formation in clone 20) might be beneficial as they add to the critical cellular mass required for potential regenerative therapies avoiding the need for excessive in vitro expansions.

(4) We can isolate the multipotent fibroblasts from regularly discarded tissues obtained during scheduled surgeries. There is absolutely no extra harm and no extra consultation time for the CLP-affected infants, which makes obtaining such cells ethically acceptable. Isolated fibroblasts can be stored in liquid nitrogen for extended times without losing any of their multipotent character (own data), and the cells can be easily and largely expanded within a short time when needed, and can be long-term passaged for at least 40 population doublings [36]. Whether the CLP lip derived-fibroblasts retain all their MSC-like characteristics over this time remains to be addressed in the future.

Altogether, our results provide evidence that CLP lip fibroblasts are a beneficial source of autologous cells for the tissue donors themselves later in life. This could be useful for the repair of the alveolar cleft in CLP patients harboring critical bone defects. Currently, the use of autologous bone grafts represents the gold standard for the repair of the alveolar clefts, as they provide all the necessary properties of osteogenicity, osteoinductivity, and osteoconductivity [75, 76]. However, collecting bone grafts involves another invasive surgery for their harvest from the chin, the iliac crest, tibia or ribs and subsequent placement into opened and prepared alveolar cleft site. Furthermore, patients undergoing this procedure might experience morbidity at the bone donor site, enhanced and unpredictable resorption rate of the graft (10–36% of the patients), and in 5% of the cases a revision surgery is also needed [77, 78]. We suggest that the use of autologous CLP lip fibroblasts, alone or in combination with a bone grafting material (e.g., deproteinized bovine bone mineral), might be beneficial in the future for patients presenting alveolar clefts. Importantly, since the reconstruction of the alveolar bone occurs at the age of 7–8 years, there would be enough time for isolating the fibroblasts at very young age, and subsequently archive them until they are needed. This approach would significantly lower the burden for CLP patients as there would be no need for bone harvesting at another site. In recent years, several human studies tried to address the repair of alveolar clefts with stem cell-based approaches including BM-MSCs. According to a recent systematic review, implantation of cells in combination with a biomaterial for the repair of the congenital cleft results in comparable bone regeneration to autogenous bone grafts [79]. Yet, to our knowledge the use of fibroblasts for neo-bone formation has never been addressed in vivo so far. Clearly, pre-clinical studies are required to test the feasibility of our hypothesis. Finally, we must acknowledge the fact that in this study we used primary fibroblasts isolated from CLP-affected individuals, whose genetic background is not known. To minimize a potential impact of a specific CLP-causing gene variant on the results presented in this work, we randomly selected five individual, non-syndromic CLP-affected tissue donors (Table 1). Working with five different donors should result in a panel of diverse genetic and/or environmental CLP risk factors. Our results show that the multipotent character as well as other cell properties are similar within the five CLP fibroblast strains, which suggests that these cells behave normally. However, we cannot fully exclude the effect of the genetic background of the individual CLP patients in controlling intrinsic cell properties, although such data in vivo are very sparse and conflicting [80–85]. Even if there might be some slight deviations in normal growth patterns in vivo, this is something that we did not observe in vitro so far comparing our cells to other healthy controls [36]. Therefore, we believe that CLP-affected patients might benefit from our approach using their autogenous fibroblasts.

## Conclusions

In summary, this is the first study demonstrating that regularly discarded CLP lip tissues are excellent sources for the isolation of multipotent fibroblasts. Specifically, we provide preliminary in vitro evidence that CLP lip-derived fibroblasts, which are abundantly present, might be an intriguing source of autologous cells for regenerative purposes, a possibility that definitely warrants future in vitro and in vivo studies.

## Supplementary Information

**Additional file 1. Fig.S1. a** Clinical picture of a CLP individual and image of the corresponding excised lip tissue biopsy before processing. Written informed consent was obtained from the parents of the individual for the publication of these images. **b** Epithelial (E) and stromal (S) cells are isolated by the explant culture system from a CLP-tissue biopsy (T) (Live Imaging) and their appearance analyzed by CV. White dashed lines in the close-ups depict the typical cell morphology of epithelial and stromal cells. IF staining of E-CAD (red) and VIM (green) confirm the epithelial or stromal-origin of the CLP outgrowths. Scale bars: 100μm (Live Imaging); 50μm (CV); 20μm (IF). Nuclei (Blue). (**c**) qPCR analysis of five CLP-derived stromal cell cultures (CLP1-CLP5, gray bars) compared to the respective reference cell line set to one (colored bars) for all the cell type-specific markers. *=p<0.05 Controls vs. CLP1-CLP5. CLP-Ep: CLP lip-derived epithelial cells; Frsk-Fb: foreskin-derived fibroblasts; HUVEC: human umbilical vein-derived endothelial cells; C2C12: myoblasts; U937: monocytes; CLP-Ad: CLP lip-derived adipocytes. **Fig.S2. a** qPCR analysis of hematopoietic markers CD31 and CD45 expression in BM-MSCs, CLP1-CLP5, WI38 cells and a reference U937 (histiocytic lymphoma-derived human monocytes) sample. *=p<0.05 U937 vs. BM-MSCs or CLP1-CLP5 or WI38. **b** BM-MSCs, CLP1-CLP5 and WI38 proliferation over a period of 7 days. **c** qPCR analysis of the basal expression of the osteogenic (yellow box,R UNX2, ALPL, SP7, SOST), adipogenic (orange box, DLK1, LPL, ADIPOQ, LEP) and chondrogenic (blue box, SOX9) markers in BM-MSCs and CLP (CLP1 or CLP3 is reported). C-ag (be): Chondrogenic-associated genes (basal expression). *=p<0.05. **Fig.S3. a** FACS analysis for the expression of the stromal marker STRO-1 in BM-MSCs, CLP and WI38 cells. Blacked dashed lines in the FACS plots indicate the threshold of unstained samples. The percentage of positive cells for each sample is reported. Gating strategy is presented in Suppl.Fig.6. **b** qPCR analysis of MSC-markers CD73, CD90 and CD105 in CLP1-CLP3 under standard (10% FCS) or reduced (0.3% FCS) serum culturing conditions. FACS analysis for CD73 expression with 10% or 0.3% FCS is reported to the right. Black dashed indicate the threshold of unstained samples. The percentage of positive cells for each sample is reported. Gating strategy is presented in Fig.S6. **c** qPCR analysis of CD106, CD146, ITGA11, IGF2, NANOG and OCT4 in BM-MSCs and WI38 under standard (10% FCS) or starving conditions.d Heatmaps reporting the results of the Luminex assay on BM-MSCs, CLP1-CLP3 and WI38 conditioned medium for 71 cytokines. (Blue: low expression; red: high expression; gray: not detected expression). Protein concentration is expressed in pg/ml. **Fig.S4. a** qPCR analysis of the MSC-markers CD106 and CD146 in BM-MSCs, parental CLP and 30 CLP-derived single cell clones. *=p<0.05. **b** qPCR analysis of DCN and S100A4 in BM-MSCs, parental CLP and 30 CLP-derived single cell clones. *=p<0.05 BM-MSCs vs. rest. **c** FACS analysis for the expression of the stromal marker STRO-1 in the parental CLP and four selected clones. Blacked dashed lines in the FACS plots indicate the threshold of unstained samples. The percentage of positive cells for each sample is reported. Gating strategy is presented in Fig.S6. **Fig.S5.** Images of the full-length blots of all immunoblotting experiments. Molecular weights are indicated to the right of the blots. Detected proteins are indicated on the bottom of each blot. Note that sometime membranes were reprobed with different antibodies. **a** Fig.1c and Fig.1e, **b** Fig.6c. **Fig.S6.** Gating strategies of all FACS experiments. The percentage of analyzed cells is reported. **Fig.S5.** Fig.2b, **b** Fig.3b and Suppl.Fig.3b, **c** Suppl.Fig.3a **d** Fig.4b, **e** Fig.5b (histograms for CD106 and CD146 expression are also reported), (**f**) Fig.6b and Fig.S4c. **Table S1.** qPCR primer sequences. **Table S2**. Summary of pro- and anti-inflammatory cytokines quantification in BM-MSCs, CLP1-CLP3 and WI38 conditioned medium by multiplex array. Data should be read as pg/ml. n.d.=not detected.

## Data Availability

The datasets generate and/or analyzed during the current study are available from the corresponding author on reasonable request.

## Abbreviations

CLP: Cleft lip and palate
MSCs: Mesenchymal stem cells
ECM: Extracellular matrix
BM-MSCs: Bone marrow-derived mesenchymal cells
FSP1: Fibroblast-specific protein 1
IHC: Immunohistochemistry
RT: Room temperature
H&E: Hematoxylin–eosin
DMEM: Dulbecco’s modified Eagle’s medium
FCS: Fetal calf serum
PBS: Phosphate-buffered saline
HUVEC: Human umbilical vein endothelial cells
CLP-Ad: Adipocytes
CV: Crystal violet
ddH_2_O: Double-distilled water
cc: Cell circularity
IF: Immunofluorescence
qPCR: Quantitative real-time polymerase chain reaction (qPCR)
CM: Conditioned media
TBS-T: Tris-buffered saline Tween-20
FACS: Flow cytometry
αMEM: α-Minimal essential medium
ARS: Alizarin Red S
ORO: Oil Red O
AB: Alcian blue
E-CAD: E-Cadherin
VIM: Vimentin
PECAM1: Platelet endothelial cell adhesion molecule-1
SMM: Smooth muscle myosin
Frsk-Fb: Foreskin fibroblasts
DCN: Decorin
PDGFRα: Platelet-derived growth factor receptor-α
CLP-Ep: Epithelial cells
FN: Fibronectin
IL6: Interleukin-6
IL-1ß: Interleukin-1ß
IL-12p40: Interleukin-12 ß subunit
CXCL5: Chemokine 5 C-X-C motif
SCF: Stem cell factor
TNFα: Tumor necrosis factor α
TNFß: Tumor necrosis factor ß
SDF-1: Stromal cell-derived factor-1
IL-1RA: Interleukin-1 receptor antagonist
TLRs: Toll-like receptors
CCL5: C-C motif chemokine ligand 5
PTGS2: Prostaglandin–endoperoxide synthase 2
CXCL8: Interleukin-8
CFU: Colony-forming unit assay
NCCs: Neural crest cells
