# Supplementary material for: Discovery and characterization of heterogeneous and multipotent fibroblast populations isolated from excised cleft lip tissue
Source: Stem Cell Res Ther. 2022 Sep 8;13:469. doi: 10.1186/s13287-022-03154-x (PMC9461253; doi:10.1186/s13287-022-03154-x)
Supplement: Supplementary file 1 — Additional file 1. Fig.S1. a Clinical picture of a CLP individual and image of the corresponding excised lip tissue biopsy before processing. Written informed consent was obtained from the parents of the individual for the publication of these images. b Epithelial (E) and stromal (S) cells are isolated by the explant culture system from a CLP-tissue biopsy (T) (Live Imaging) and their appearance analyzed by CV. White dashed lines in the close-ups depict the typical cell morphology of epithelial and stromal cells. IF staining of E-CAD (red) and VIM (green) confirm the epithelial or stromal-origin of the CLP outgrowths. Scale bars: 100μm (Live Imaging); 50μm (CV); 20μm (IF). Nuclei (Blue). (c) qPCR analysis of five CLP-derived stromal cell cultures (CLP1-CLP5, gray bars) compared to the respective reference cell line set to one (colored bars) for all the cell type-specific markers. *=p<0.05 Controls vs. CLP1-CLP5. CLP-Ep: CLP lip-derived epithelial cells; Frsk-Fb: foreskin-derived fibroblasts; HUVEC: human umbilical vein-derived endothelial cells; C2C12: myoblasts; U937: monocytes; CLP-Ad: CLP lip-derived adipocytes. Fig.S2. a qPCR analysis of hematopoietic markers CD31 and CD45 expression in BM-MSCs, CLP1-CLP5, WI38 cells and a reference U937 (histiocytic lymphoma-derived human monocytes) sample. *=p<0.05 U937 vs. BM-MSCs or CLP1-CLP5 or WI38. b BM-MSCs, CLP1-CLP5 and WI38 proliferation over a period of 7 days. c qPCR analysis of the basal expression of the osteogenic (yellow box,R UNX2, ALPL, SP7, SOST), adipogenic (orange box, DLK1, LPL, ADIPOQ, LEP) and chondrogenic (blue box, SOX9) markers in BM-MSCs and CLP (CLP1 or CLP3 is reported). C-ag (be): Chondrogenic-associated genes (basal expression). *=p<0.05. Fig.S3. a FACS analysis for the expression of the stromal marker STRO-1 in BM-MSCs, CLP and WI38 cells. Blacked dashed lines in the FACS plots indicate the threshold of unstained samples. The percentage of positive cells for each sample is reported. Gating strat [file 13287_2022_3154_MOESM1_ESM.docx]

**Supplementary Materials**

**Discovery and characterization of heterogeneous and multipotent fibroblast populations isolated from excised cleft lip tissue**

**Stem Cell Research and Therapy**

Ludovica Parisi, Silvia Rihs, Giorgio C. La Scala, Isabelle Schnyder, Christos Katsaros, Martin Degen


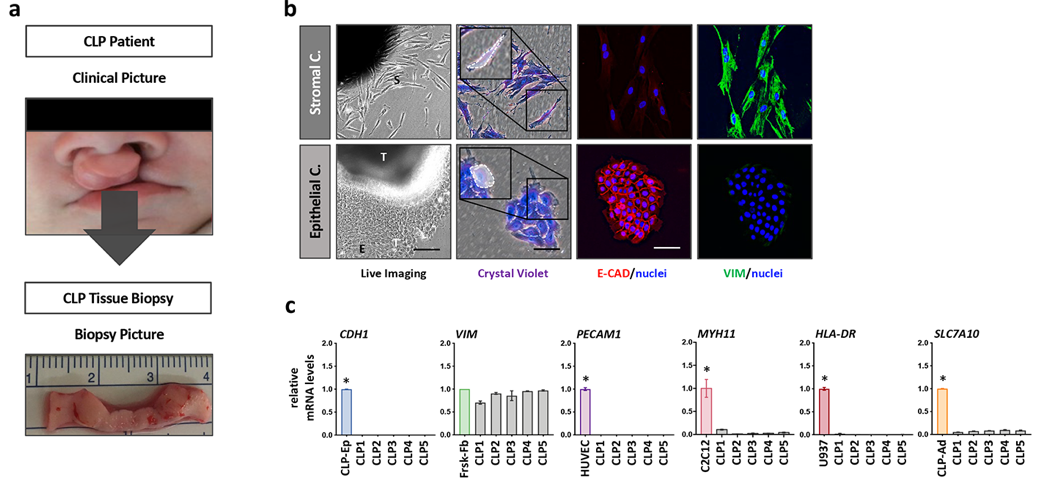


**Suppl.Fig.1** (**a**) Clinical picture of a CLP individual and image of the corresponding excised lip tissue biopsy before processing. Written informed consent was obtained from the parents of the individual for the publication of these images. (**b**) Epithelial (E) and stromal (S) cells are isolated by the explant culture system from a CLP-tissue biopsy (T) (Live Imaging) and their appearance analyzed by CV. White dashed lines in the close-ups depict the typical cell morphology of epithelial and stromal cells. IF staining of E-CAD (red) and VIM (green) confirm the epithelial or stromal-origin of the CLP outgrowths. Scale bars: 100µm (Live Imaging); 50µm (CV); 20µm (IF). Nuclei (Blue). (**c**) qPCR analysis of five CLP-derived stromal cell cultures (CLP1-CLP5, gray bars) compared to the respective reference cell line set to one (colored bars) for all the cell type-specific markers. *=*p*<0.05 Controls vs. CLP1-CLP5. CLP-Ep: CLP lip-derived epithelial cells; Frsk-Fb: foreskin-derived fibroblasts; HUVEC: human umbilical vein-derived endothelial cells; C2C12: myoblasts; U937: monocytes; CLP-Ad: CLP lip-derived adipocytes.


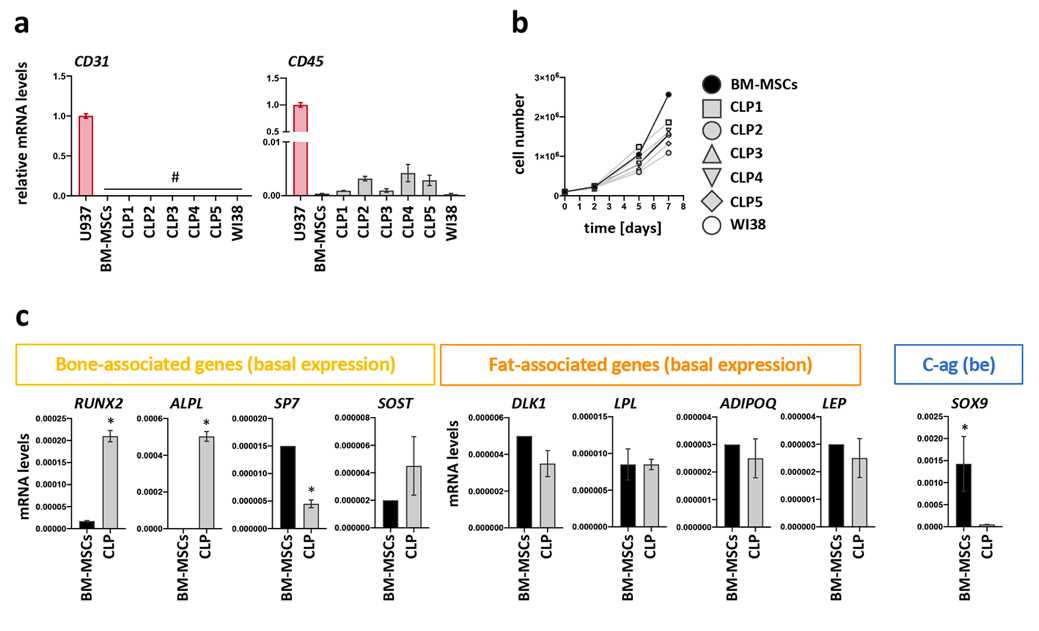


**Suppl.Fig.2:** (**a**) qPCR analysis of hematopoietic markers *CD31* and *CD45* expression in BM-MSCs, CLP1-CLP5, WI38 cells and a reference U937 (histiocytic lymphoma-derived human monocytes) sample. *=*p*<0.05 U937 vs. BM-MSCs or CLP1-CLP5 or WI38. (**b**) BM-MSCs, CLP1-CLP5 and WI38 proliferation over a period of 7 days. (**c**) qPCR analysis of the basal expression of the osteogenic (yellow box, *RUNX2, ALPL, SP7, SOST*), adipogenic (orange box, *DLK1, LPL, ADIPOQ, LEP*) and chondrogenic (blue box, *SOX9*) markers in BM-MSCs and CLP (CLP1 or CLP3 is reported). C-ag (be): Chondrogenic-associated genes (basal expression). *=*p*<0.05.


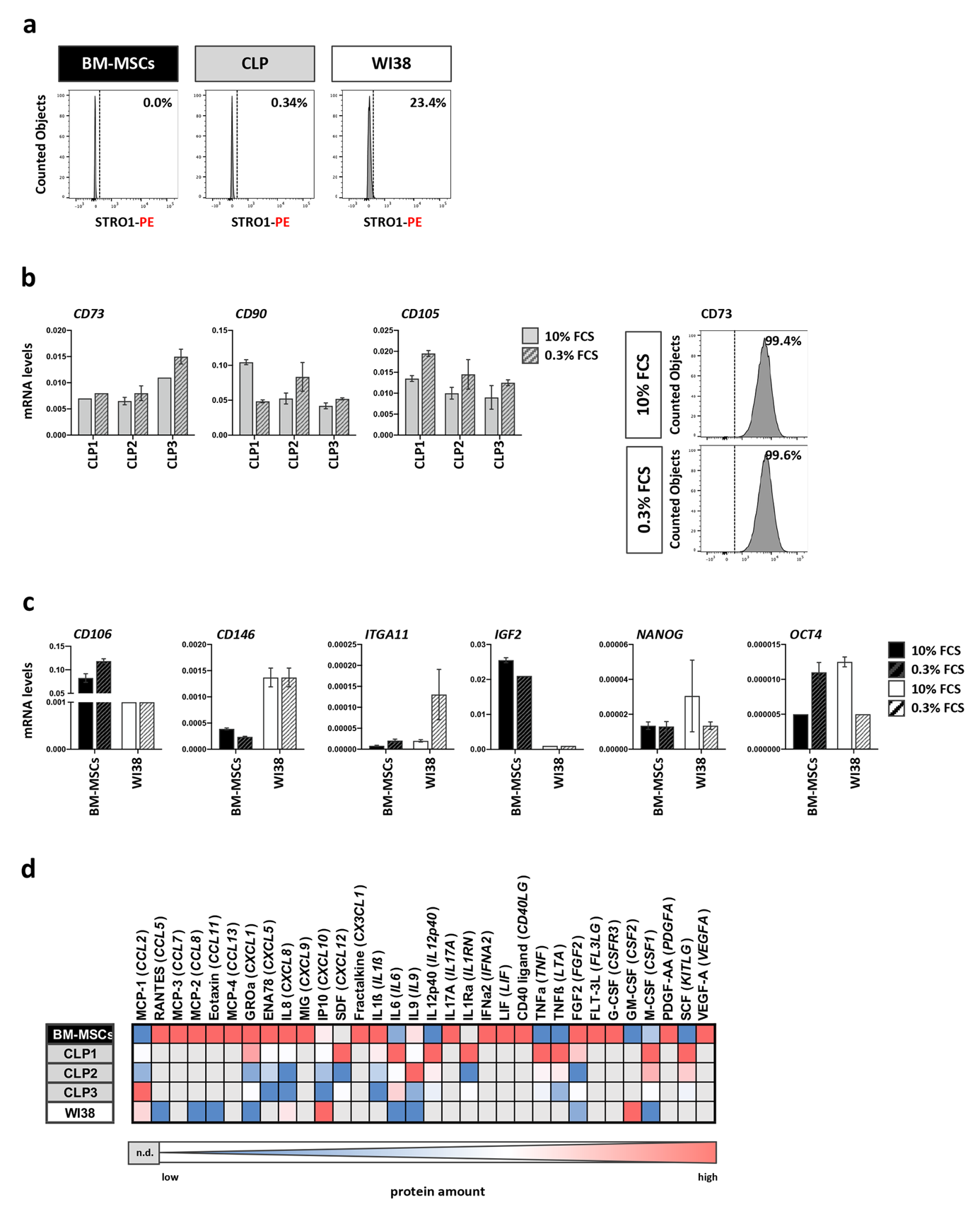


**Suppl.Fig.3:** (**a**) FACS analysis for the expression of the stromal marker STRO-1 in BM-MSCs, CLP and WI38 cells. Blacked dashed lines in the FACS plots indicate the threshold of unstained samples. The percentage of positive cells for each sample is reported. Gating strategy is presented in **Suppl.Fig.6**. (**b**) qPCR analysis of MSC-markers *CD73*, *CD90* and *CD105* in CLP1-CLP3 under standard (10% FCS) or reduced (0.3% FCS) serum culturing conditions. FACS analysis for CD73 expression with 10% or 0.3% FCS is reported to the right. Black dashed indicate the threshold of unstained samples. The percentage of positive cells for each sample is reported. Gating strategy is presented in **Suppl.Fig.6**. (**c**) qPCR analysis of *CD106*, *CD146*, *ITGA11*, *IGF2*, *NANOG* and *OCT4* in BM-MSCs and WI38 under standard (10% FCS) or starving conditions. (**d**) Heatmaps reporting the results of the Luminex assay on BM-MSCs, CLP1-CLP3 and WI38 conditioned medium for 71 cytokines. (Blue: low expression; red: high expression; gray: not detected expression). Protein concentration is expressed in pg/ml.


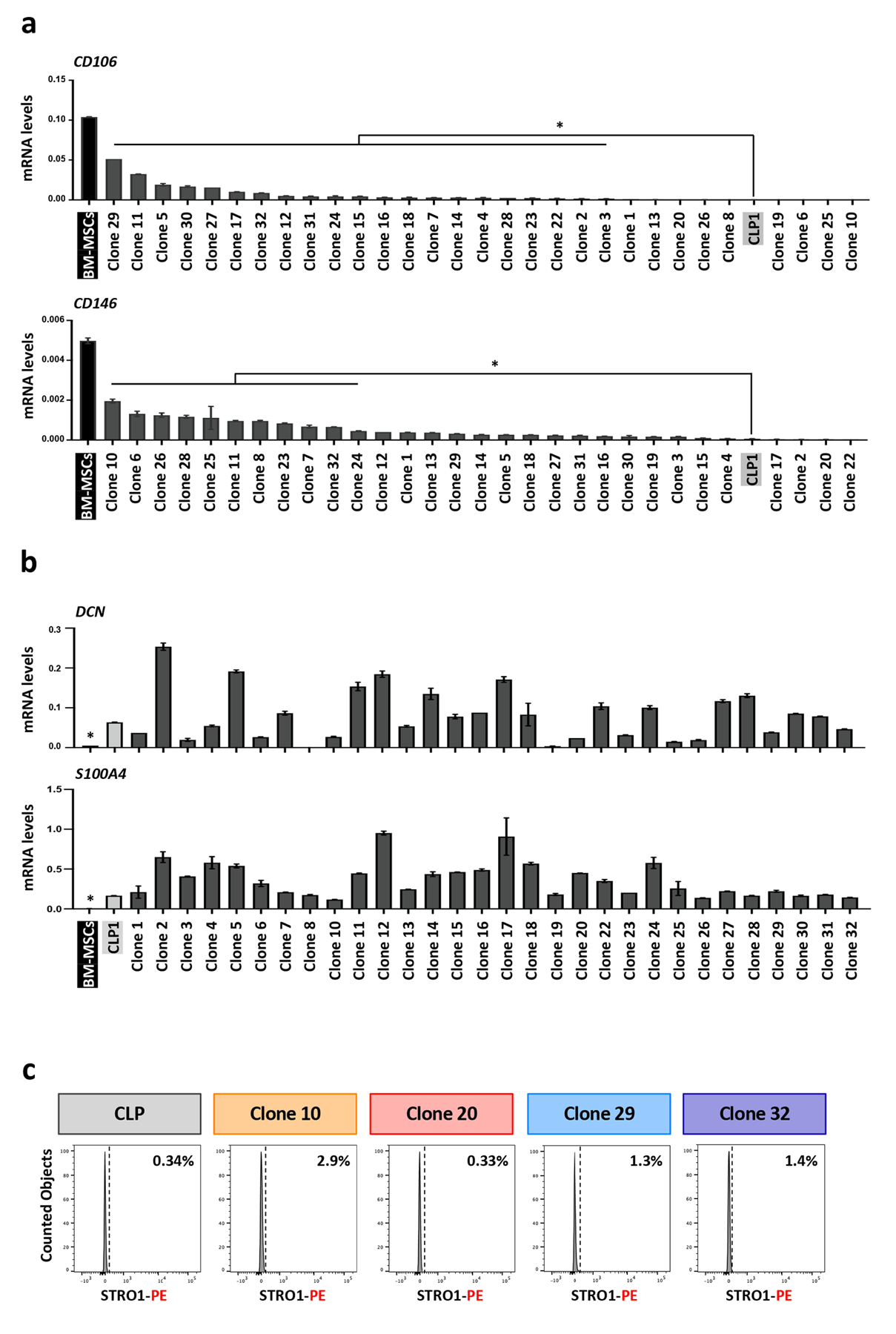


**Suppl.Fig.4** (**a**) qPCR analysis of the MSC-markers *CD106* and *CD146* in BM-MSCs, parental CLP and 30 CLP-derived single cell clones. *=p<0.05. (**b**) qPCR analysis of *DCN* and *S100A4* in BM-MSCs, parental CLP and 30 CLP-derived single cell clones. *=p<0.05 BM-MSCs vs. rest. (**c**) FACS analysis for the expression of the stromal marker STRO-1 in the parental CLP and four selected clones. Blacked dashed lines in the FACS plots indicate the threshold of unstained samples. The percentage of positive cells for each sample is reported. Gating strategy is presented in **Suppl.Fig.6**.

**
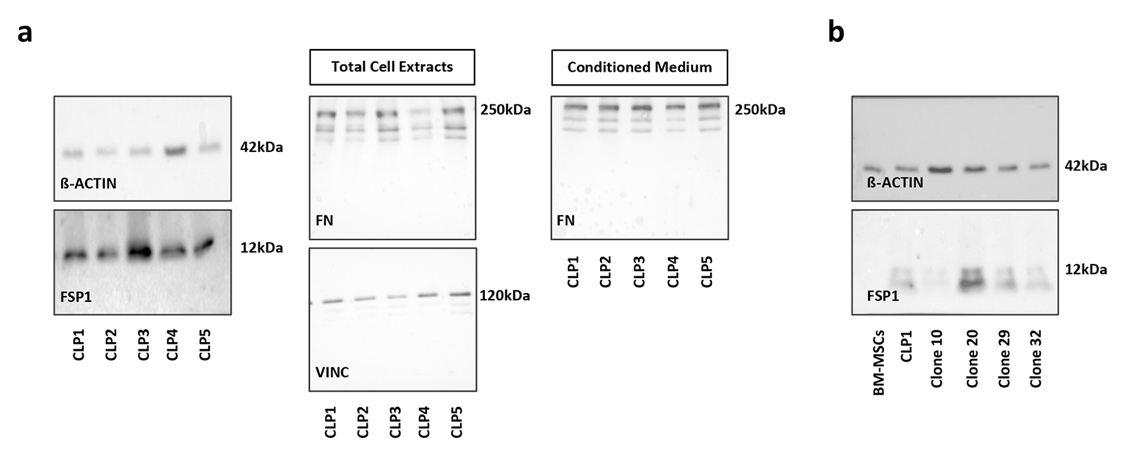
**

**Suppl.Fig.5** Images of the full-length blots of all immunoblotting experiments. Molecular weights are indicated to the right of the blots. Detected proteins are indicated on the bottom of each blot. Note that sometime membranes were reprobed with different antibodies. (**a**) Fig.1c and Fig.1e, (**b**) Fig.6c.


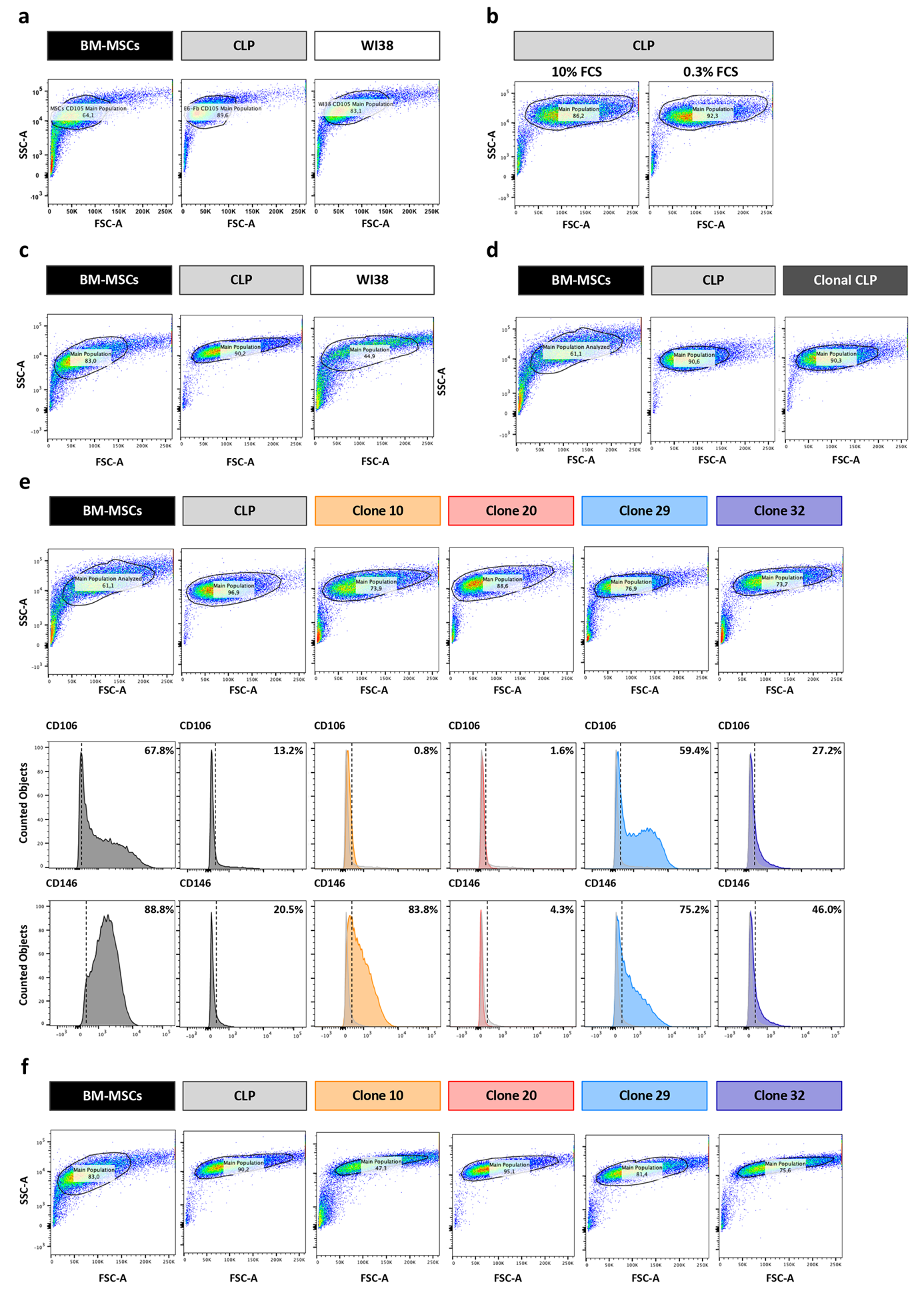


**Suppl.Fig.6** Gating strategies of all FACS experiments. The percentage of analyzed cells is reported. (**a**) Fig.2b, (**b**) Fig.3b and Suppl.Fig.3b, (**c**) Suppl.Fig.3a (**d**) Fig.4b, (**e**) Fig.5b (histograms for CD106 and CD146 expression are also reported), (**f**) Fig.6b and Suppl.Fig.4c.

**Suppl.Table1** qPCR primer sequences

| **gene** | **Forward (5'->3')** | **Reverse (5'->3')** |
| --- | --- | --- |
| ***CDH1*** | AGAACGCATTGCCACATACACT | TCTGATCGGTTACCGTGATCAA |
| ***VIM*** | TGTCCAAATCGATGTGGATGTTTC | TTGTACCATTCTTCTGCCTCCTG |
| ***PECAM*** | AACAGTGTTGACATGAAGAGCC | TGTAAAACAGCACGTCATCCTT |
| ***MYH11*** | GTCCAGGAGATGAGGCAGAAAC | GTCTGCGTTCTCTTTCTCCAGC |
| ***HLA-DR*** | ATACTCCGATCACCAATGTACCT | GACTGTCTCTGACACTCCTGT |
| ***SLC7A10*** | TCTAAAGCGGGGTAGGAAGAA | GTCCTGTCCCTCTCTTGTGTA |
| ***DCN*** | GCTCTCCTACATCCGCATTGCT | GTCCTTTCAGGCTAGCTGCATC |
| ***PDGFRa*** | GACTTTCGCCAAAGTGGAGGAG | AGCCACCGTGAGTTCAGAACGC |
| ***S100A4*** | CCACAAGTACTCGGGCAAAG | GTCCCTGTTGCTGTCCAAGT |
| ***CD73*** | CCAGTACCAGGGCACTATCTG | TGGCTCGATCAGTCCTTCCA |
| ***CD90*** | ATCGCTCTCCTGCTAACAGTC | CTCGTACTGGATGGGTGAACT |
| ***CD105*** | TGCACTTGGCCTACAATTCCA | AGCTGCCCACTCAAGGATCT |
| ***RUNX2*** | CCGCCTCAGTGATTTAGGGC | GGGTCTGTAATCTGACTCTGTCC |
| ***ALPL*** | ACTGGTACTCAGACAACGAGAT | ACGTCAATGTCCCTGATGTTATG |
| ***SP7*** | CCTCTGCGGGACTCAACAAC | AGCCCATTAGTGCTTGTAAAGG |
| ***SOST*** | GGAGCTGGAGAACAACAAGACC | TCACGTAGCGGGTGAAGTGCAG |
| ***DLK1*** | CCCCAAAATGGATTCTGCGAGG | GGTTCTCCACAGAGTCCGTGAA |
| ***LPL*** | ACGGCATGTGAATTCTGTGA | GGATGTGCTATTTGGCCACT |
| ***ADIPOQ*** | TGCTGGGAGCTGTTCTACTG | TACTCCGGTTTCACCGATGTC |
| ***LEP*** | TGCCTTCCAGAAACGTGATCC | CTCTGTGGAGTAGCCTGAAGC |
| ***SOX9*** | AGCGAACGCACATCAAGAC | CTGTAGGCGATCTGTTGGGG |
| ***CD106*** | TTTGACAGGCTGGAGATAGACT | TCAATGTGTAATTTAGCTCGGCA |
| ***CD146*** | GGTCGCTACCTGTGTAGGGA | TGGACCCGGTTCTTCTCCT |
| ***ITGA11*** | GACCTACATGGACATCGTCATTG | ATCTTCGCCATACTGCACAAC |
| ***IGF2*** | GTGGCATCGTTGAGGAGTG | CACGTCCCTCTCGGACTTG |
| ***NANOG*** | AAGGTCCCGGTCAAGAAACAG | CTTCTGCGTCACACCATTGC |
| ***OCT4*** | CTTGAATCCCGAATGGAAAGGG | GTGTATATCCCAGGGTGATCCTC |
| ***TLR3*** | GCGCTAAAAAGTGAAGAACTGGAT | GCTGGACATTGTTCAGAAAGAGG |
| ***TLR4*** | CCCTGAGGCATTTAGGCAGCTA | AGGTAGAGAGGTGGCTTAGGCT |
| ***CCL5*** | CCTGCTGCTTTGCCTACATTGC | ACACACTTGGCGGTTCTTTCGG |
| ***PTGS2*** | TCCTAACCCTTTTGTCGCCTG | CGCTTCCCAGAGGATCTGC |
| ***IL6*** | ACTCACCTCTTCAGAACGAATTG | CCATCTTTGGAAGGTTCAGGTTG |
| ***CXCL8*** | GAGAGTGATTGAGAGGTGGACCAC | CACAACCCTCTGCAXCCCAGTTT |
| ***CD45*** | ACCACAAGTTTACTAACGCAAGT | TTTGAGGGGGATTCCAGGTAAT |

**Suppl.Table 2** Summary of pro- and anti-inflammatory cytokines quantification in BM-MSCs, CLP1-CLP3 and WI38 conditioned medium by multiplex array. Data should be read as pg/ml. n.d.=not detected.

|  | **BM-MSCs** | **CLP1** | **CLP2** | **CLP3** | **WI38** |
| --- | --- | --- | --- | --- | --- |
| **sCD40L** | 1,60 | n.d. | n.d. | n.d. | n.d. |
| **EGF** | n.d. | n.d. | n.d. | n.d. | n.d. |
| **Eotaxin** | 1,17 | n.d. | n.d. | n.d. | 0,28 |
| **FGF-2** | 15,92 | 8,30 | 2,90 | 4,16 | 3,30 |
| **FLT-3L** | 0,12 | n.d. | n.d. | n.d. | n.d. |
| **Fractalkine** | 4,57 | n.d. | n.d. | n.d. | n.d. |
| **G-CSF** | 258,40 | n.d. | n.d. | n.d. | n.d. |
| **GM-CSF** | 1,97 | n.d. | n.d. | n.d. | 0,37 |
| **GROa** | 313,51 | 199,92 | 8,62 | 24,66 | 1,81 |
| **IFN-a2** | 1,21 | n.d. | n.d. | n.d. | n.d. |
| **IFNy** | n.d. | n.d. | n.d. | n.d. | n.d. |
| **IL-1a** | n.d. | n.d. | n.d. | n.d. | n.d. |
| **IL-1ß** | 1,06 | 0,84 | 0,90 | 0,73 | n.d. |
| **IL-1RA** | n.d. | 0,55 | 1,39 | 1,25 | n.d. |
| **IL-2** | n.d. | n.d. | n.d. | n.d. | n.d. |
| **IL-3** | n.d. | n.d. | n.d. | n.d. | n.d. |
| **IL-4** | n.d. | n.d. | n.d. | n.d. | n.d. |
| **IL-5** | n.d. | n.d. | n.d. | n.d. | n.d. |
| **IL-6** | 88,49 | 1397,95 | 233,14 | 573,35 | 23,23 |
| **IL-7** | n.d. | n.d. | n.d. | n.d. | n.d. |
| **IL-8** | 827,05 | 124,60 | 15,47 | 19,25 | 245,98 |
| **IL-9** | 0,57 | 0,53 | 0,74 | 0,44 | 0,40 |
| **IL-10** | n.d. | n.d. | n.d. | n.d. | n.d. |
| **IL-12p40** | 0,87 | 1,55 | 1,25 | 0,51 | n.d. |
| **IL-12p70** | n.d. | n.d. | n.d. | n.d. | n.d. |
| **IL-13** | n.d. | n.d. | n.d. | n.d. | n.d. |
| **IL-15** | n.d. | n.d. | n.d. | n.d. | n.d. |
| **IL-17A** | 0,20 | n.d. | n.d. | n.d. | n.d. |
| **IL-17E/IL-25** | n.d. | n.d. | n.d. | n.d. | n.d. |
| **IL-17F** | n.d. | n.d. | n.d. | n.d. | n.d. |
| **IL-18** | n.d. | n.d. | n.d. | n.d. | n.d. |
| **IL-22** | n.d. | n.d. | n.d. | n.d. | n.d. |
| **IL-27** | n.d. | n.d. | n.d. | n.d. | n.d. |
| **IP-10** | 1,00 | 0,68 | 0,47 | 0,26 | 3,37 |
| **MCP-1** | 73,77 | 88,10 | 79,18 | 97,01 | 90,45 |
| **MCP-3** | 2,78 | n.d. | n.d. | n.d. | n.d. |
| **M-CSF** | 6,35 | 9,26 | 2,15 | 7,33 | 5,11 |
| **MDC** | n.d. | n.d. | n.d. | n.d. | n.d. |
| **MIG/CXCL9** | 1,34 | n.d. | n.d. | n.d. | n.d. |
| **MIP-1a** | n.d. | n.d. | n.d. | n.d. | n.d. |
| **MIP-1ß** | n.d. | n.d. | n.d. | n.d. | n.d. |
| **PDGF-AA** | 52,75 | n.d. | n.d. | n.d. | n.d. |
| **PDGF-AB/BB** | n.d. | n.d. | n.d. | n.d. | n.d. |
| **RANTES** | 3,19 | n.d. | n.d. | n.d. | 0,61 |
| **TGFa** | n.d. | n.d. | n.d. | n.d. | n.d. |
| **TNFa** | 1,06 | 2,08 | 1,57 | 1,50 | n.d. |
| **TNFß** | 0,57 | 1,19 | 0,74 | 0,61 | n.d. |
| **VEGF-A** | 2,87 | n.d. | n.d. | n.d. | n.d. |
| **6CKine** | n.d. | n.d. | n.d. | n.d. | n.d. |
| **BCA-1** | n.d. | n.d. | n.d. | n.d. | n.d. |
| **CTACK** | n.d. | n.d. | n.d. | n.d. | n.d. |
| **ENA-78** | 2053,36 | 9,63 | 6,03 | 2,43 | n.d. |
| **Eotaxin-2** | n.d. | n.d. | n.d. | n.d. | n.d. |
| **Eotaxin-3** | n.d. | n.d. | n.d. | n.d. | n.d. |
| **I-309** | n.d. | n.d. | n.d. | n.d. | n.d. |
| **IL-16** | n.d. | n.d. | n.d. | n.d. | n.d. |
| **IL-20** | n.d. | n.d. | n.d. | n.d. | n.d. |
| **IL-21** | n.d. | n.d. | n.d. | n.d. | n.d. |
| **IL-23** | n.d. | n.d. | n.d. | n.d. | n.d. |
| **IL-28A** | n.d. | n.d. | n.d. | n.d. | n.d. |
| **IL-33** | n.d. | n.d. | n.d. | n.d. | n.d. |
| **LIF** | 11,18 | n.d. | n.d. | n.d. | n.d. |
| **MCP-2** | 5,66 | n.d. | n.d. | n.d. | 3,66 |
| **MCP-4** | 12,39 | n.d. | n.d. | n.d. | n.d. |
| **MIP-1d** | n.d. | n.d. | n.d. | n.d. | n.d. |
| **SCF** | 1,13 | 2,09 | 1,98 | 1,86 | n.d. |
| **SDF-1a+ß** | n.d. | 696,13 | 477,78 | 587,76 | n.d. |
| **TARC** | n.d. | n.d. | n.d. | n.d. | n.d. |
| **TPO** | n.d. | n.d. | n.d. | n.d. | n.d. |
| **TRAIL** | n.d. | n.d. | n.d. | n.d. | n.d. |
| **TSLP** | n.d. | n.d. | n.d. | n.d. | n.d. |
